# Microwave Field Enhancement at Metal‐Electrolyte Interfaces Enables Rapid Growth of Fe‐Ni_3_S_2_ on Nickel Foam for Alkaline Oxygen Evolution

**DOI:** 10.1002/smsc.70267

**Published:** 2026-04-04

**Authors:** Dongbeom Kim, Sang‐Mun Jung, Byung‐Jo Lee, Sangmin Ryu, Wonjoon Choi, Yoonsun Choi, Geonwoo Kim, Ji Hye Kwak, Sunshin Jung, Yong‐Tae Kim, Unyong Jeong

**Affiliations:** ^1^ Department of Materials Science and Engineering Pohang University of Science and Technology (POSTECH) Pohang Republic of Korea; ^2^ Electrical Environment Research Center Korea Electrotechnology Research Institute Changwon Republic of Korea

**Keywords:** alkaline water electrolyzer, field enhancement effect, microwave heating, oxygen evolution reaction

## Abstract

High‐performance oxygen evolution reaction (OER) catalysts must combine low overpotential, fast electron/ion transport, low‐cost scale‐up production, and long‐term durability under alkaline operation. Although Fe‐doped Ni_3_S_2_ is considered a catalyst satisfying the conditions, rapid uniform growth of Fe‐Ni_3_S_2_ on metallic Ni supports remains challenging. Herein, we circumvent this long‐standing obstacle by harnessing a localized microwave field enhancement effect at the electrode–electrolyte interface. This strategy enables rapid (15 min) and low‐power (75 W) growth of a highly crystalline Fe‐Ni_3_S_2_ catalyst directly on the Ni foam substrate. The as‐synthesized catalyst shows exceptional OER performance, exhibiting a low overpotential of 270 mV at 100 mA cm^−2^ and outstanding durability for over 100 h. Moreover, the successful operation of an anion exchange membrane water electrolysis device demonstrates its practical feasibility. This study not only unveils a superior OER electrocatalyst but also establishes the principle of microwave field enhancement as a new and powerful paradigm for the energy‐efficient synthesis of functional materials directly on metallic platforms.

## Graphical abstract

A rapid microwave‐assisted interfacial synthesis strategy enables the conformal growth of Fe‐Ni_3_S_2_ on Nickel foam. By harnessing localized microwave field enhancement at the electrode‐electrolyte interface, this approach facilitates efficient catalyst formation under low‐power conditions. This study establishes a versatile paradigm for energy‐efficient material synthesis directly on metallic platforms, delivering high‐performance alkaline oxygen evolution.

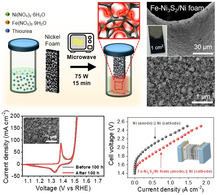

## 1 Introduction

Hydrogen is increasingly recognized as a sustainable energy carrier due to its high gravimetric energy density and minimal environmental impact, as well as its broad applicability across energy storage, chemical synthesis, and transportation [1, 2, 3, 4]. Among various production methods, water electrolysis offers high product purity, compatibility with intermittent renewable energy sources, and a fully sustainable pathway when powered by green electricity [5, 6, 7, 8, 9]. However, its efficiency is constrained by the sluggish oxygen evolution reaction (OER) at the anode—a multistep four‐electron process requiring significant overpotential [10, 11]. High‐performance OER catalysts supported on suitable current collectors must combine low overpotential, fast electron/ion transport, and long‐term durability under alkaline operation [10, 12, 13, 14]. Precious metal oxides such as IrO_2_ and RuO_2_ meet many of these criteria but suffer from high cost and inadequate long‐term stability, motivating the search for earth‐abundant alternatives [15, 16]. Ni, Fe, and Co‐based compounds—particularly hydroxides, oxides, sulfides, and selenides—have emerged as promising candidates.

Among these cost‐effective catalysts, Ni_3_S_2_ stands out for its intrinsic metallic conductivity (∼5.6 × 10^6^ S m^−1^) derived from a continuous Ni–Ni network with uniform electron density, far exceeding that of the layered double hydroxides (<10^3^ S m^−1^) where interlayer species hinder charge transport [17, 18, 19]. Fe‐doping further enhances catalytic activity by redistributing electron density near the Fermi level, lowering the d‐band energy barriers, promoting the formation of catalytically active Ni–Fe oxyhydroxides, and increasing the electrochemically active surface area [20, 21]. Despite these advantages, achieving uniform growth of Fe‐Ni_3_S_2_ on metallic Ni supports (foil, foam, felt) remains challenging. Hydrothermal synthesis is reliable, but it requires elevated temperatures (120–180°C) and long reaction times (several hours), limiting production scalability [17, 22, 23, 24, 25, 26, 27, 28]. Electrodeposition operates at low temperature but often results in poor adhesion and reproducibility [29, 30, 31]. Thus, rapid and low‐temperature synthesis of highly activated Fe‐Ni_3_S_2_ catalyst directly on Ni supports is needed for practical uses.

Microwave (MW) synthesis offers a promising synthetic pathway, providing rapid volumetric heating, high energy efficiency, and rapid completion of reaction (typically within minutes) [32, 33, 34]. More importantly, MW irradiation can enhance adhesion of the catalytic crystals on the support due to localized interfacial heating by enhanced MW fields. It is well established that bulk metals—metallic structures like foils and foams that are much thicker than the MW skin depth—are not appropriate because of their high conductivity, which causes significant reflection of the incident MWs [35, 36]. Any remaining energy is confined to a shallow surface layer, a phenomenon known as the skin effect, leading to negligible internal electromagnetic fields and thus minimal bulk heating [37]. This conventional knowledge has limited the application of MW heating for synthesis of catalysts directly on metallic supports. Recent studies have revealed that strong electric fields at metal–dielectric interfaces can create localized hotspots, enabling the direct synthesis of nanostructured materials on metallic supports [38, 39]. Despite this potential, MW‐assisted synthesis of high‐performance electrocatalysts on metallic supports remains largely unexplored. Furthermore, controlled synthesis to achieve uniform electrocatalysts on porous metal supports has rarely been investigated.

In this study, we realize one‐step growth of uniform Fe‐Ni_3_S_2_ catalysts directly on a nickel foam by employing the interfacial field enhancement of MW across the interconnected network of the nickel foam. This strategy enables rapid synthesis of high‐performance catalysts in porous metal supports under exceptionally mild conditions (75 W for 15 min) without external heating, which is mostly desired for practical catalyst production. The resulting electrode achieves 100 mA cm^−2^ at 1.50 V vs. reversible hydrogen electrode (RHE) (overpotential ≈270 mV) in alkaline electrolyte, with stability exceeding 100 h. In a practical alkaline water electrolyzer (AWE) operated at 400 mA cm^−2^, stable performance is maintained for 72 h. This work demonstrates that tailoring substrate morphology to optimize interfacial MW field distribution enables a scalable, energy‐efficient route for producing high‐performance catalysts directly onto metallic current collectors.

## 2 Results and Discussion

### 2.1 Localized Heating at the Metal‐Solution Interface by the Interfacial MW Field Enhancement

The localized heating via low‐power MW irradiation was investigated by examining the MW interaction with each component used in the synthesis. For this study, a custom‐built single‐mode MW reactor operating at a low power (50 W) was utilized. MW absorption was precisely quantified by measuring the reflected power, while the real‐time surface temperature of each component was monitored using an IR sensor (the detailed configuration of the equipment used in this study is provided in Supporting Information, Figure S1).

**FIGURE 1:**
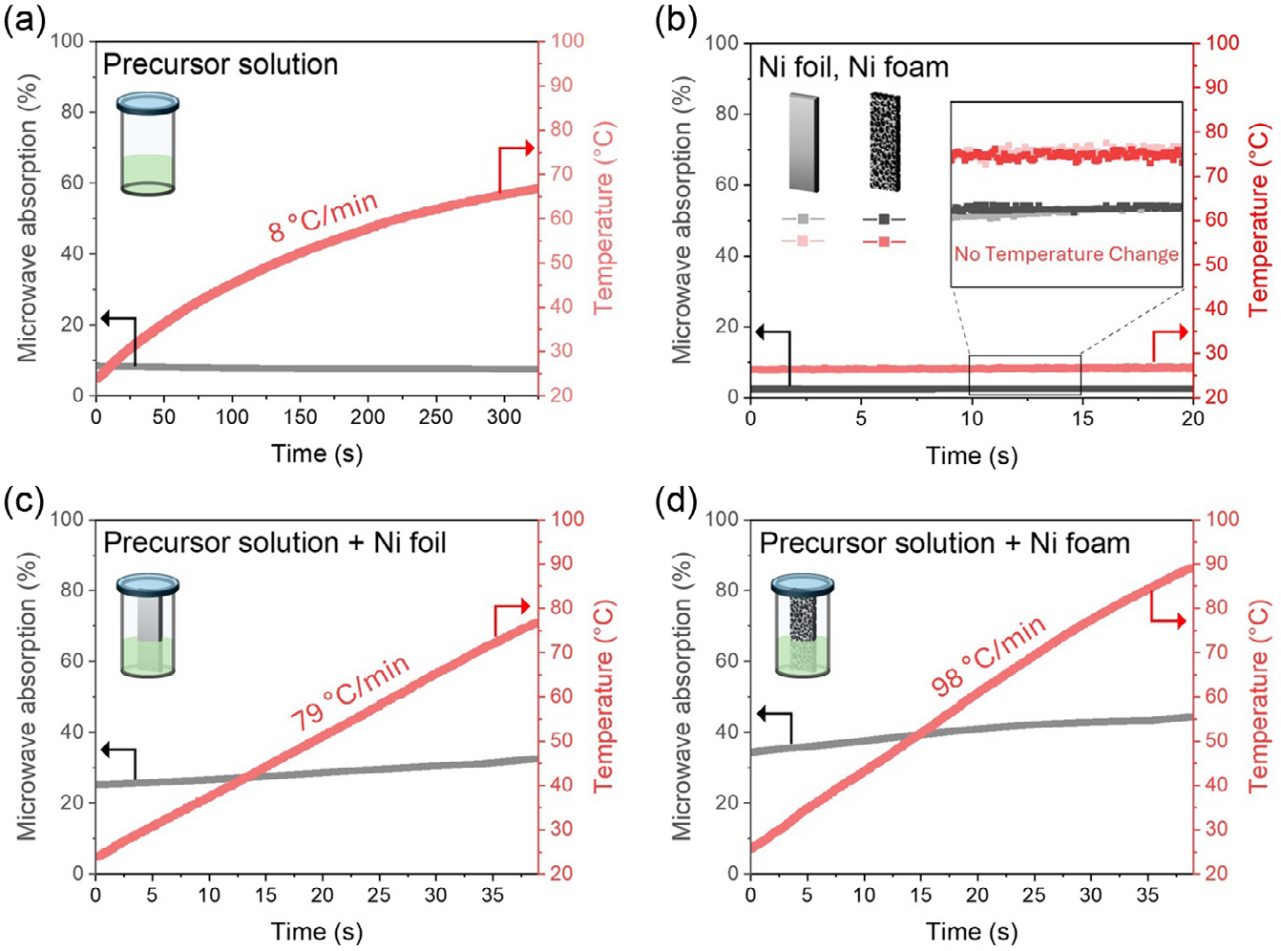
MW absorption rates of the Ni foil and Ni foam and corresponding temperature profiles increases: (a) precursor solution, (b) Ni foil and Ni foam in the air, (c) Ni foil immersed in the precursor solution, and (d) Ni foam immersed in the precursor solution.

Figure 1 illustrates the MW absorption rates for four different scenarios and the corresponding temperature profiles, which were investigated to understand the heating source by MW absorption. The standard precursor solution used for these tests consisted of 30 mM Ni(NO_3_)_2_ · 6H_2_O, 10 mM Fe(NO_3_)_3_·9H_2_O, and 720 mM thiourea (the optimization of the solution is detailed in the following section). As shown in Figure 1a, this precursor solution alone exhibited an average MW absorption of 8%, resulting in a heating rate of approximately 8°C/min and reaching a temperature of 65°C after 300 s. When the bulk Ni foil and Ni foam were irradiated in the absence of the solution (in air), they showed no MW absorbance, thus no temperature increase (Figure 1b). These results are well aligned with the conventional understanding that bulk metals are MW reflectors, thus unsuitable for volumetric heating. A dramatic synergistic effect was observed, however, when the metallic substrates were immersed in the precursor solution. Upon introducing the Ni foil into the solution (Figure 1c), the overall MW absorption of the system significantly increased to an average of ∼30%. This led to a remarkably rapid temperature increase, with a heating rate of approximately 79°C/min. The effect was more significant with the Ni foam immersed in the precursor solution (Figure 1d). In this case, the MW absorption jumped to ∼40%, resulting in an exceptionally high heating rate of approximately 98°C/min, reaching 90°C in 40 s of MW irradiation. These findings suggest a heating mechanism: neither the metallic substrate nor the precursor solution alone efficiently absorbs low‐power MW energy, and MW field is largely enhanced at the interface between a conductive metallic body and a dielectric precursor solution.

**FIGURE 2:**
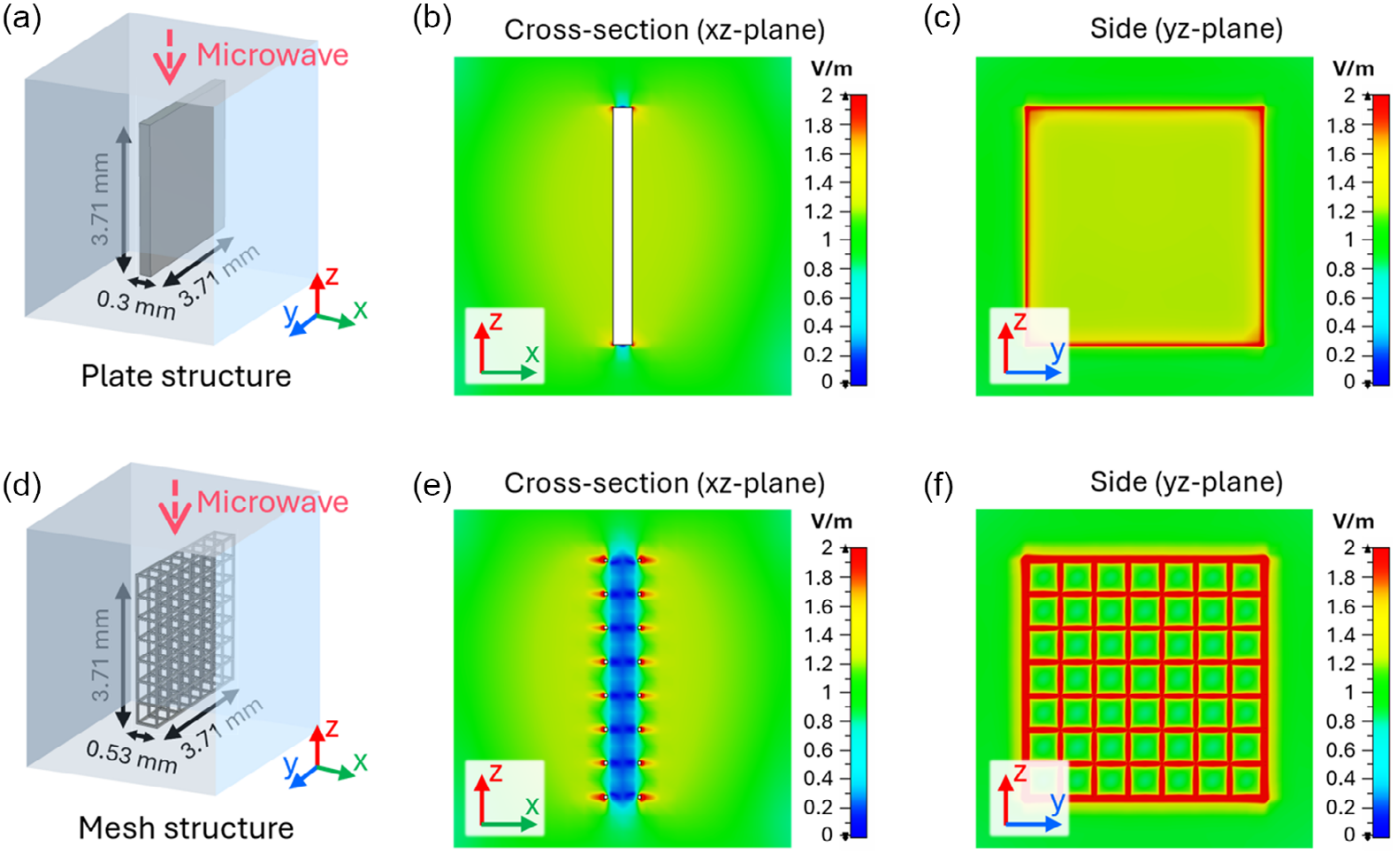
Comparison of simulated electric field intensity distributions for the Ni foil (a–c) and the Ni mesh (d–f) immersed in the precursor solution: simulation models (a,d), cross‐sectional view for the electric field distribution (b,e), and the side view electric field distribution (c,f).

To further investigate the synergistic heating and to visualize the spatial distribution of the electric field, we performed simulations using CST Studio Suite. The solution environment for the simulation was established by measuring the dielectric constant of the precursor solution (Table S1). Figure 2a shows the simulation model for an Ni foil immersed in the precursor solution. The Ni foil was assumed to be a Ni plate with a thickness of 0.3 mm. The electric field was concentrated along its sharp edges and corners, as seen in the cross‐sectional view (*xz* plane, Figure 2b) and the side view (*yz* plane, Figure 2c). Localization of the field at these geometric singularities explains the enhanced heating observed experimentally. Figure 2d presents the simulation model for the Ni foam immersed in the precursor solution. The porous structure of the Ni foam was designed as a 3D mesh structure. Contrary to the Ni foil, the 3D Ni mesh frame (ligaments and junctions) throughout the entire porous volume induced an intensely localized electric field, creating a dense network of MW fields, as shown from the cross‐sectional view (Figure 2e) and the side view (Figure 2f). This amplified electric field induces dielectric loss, which is converted into heat. The induced dielectric loss distributions are visualized in Figure S2. Collectively, the experimental heating profiles and simulation results provide compelling evidence for the localized MW field enhancement at the interface between the conductive metallic substrate and the precursor solution. While the Ni foil has field enhancement primarily at its edges, the 3D porous structure of the Ni foam creates an intense network of localized field enhancement, which leads to exclusive heating at the interface.

The dramatic increase in microwave (MW) absorption within the combined metal‐solution system is attributed to a synergistic coupling between the electromagnetic field concentrator (Ni) and the dielectric energy dissipater (precursor solution). Bulk Ni substrates act as near‐perfect MW reflectors due to their high electrical conductivity, which prevents the penetration of electromagnetic fields into the bulk metal material. Simultaneously, while the precursor solution is a moderate absorber with a measured dielectric loss factor (ε″) of approximately 15.95 (ε′ ≈ 76.58, tan δ ≈ 0.21), it lacks a mechanism to concentrate the electric field (*E*), resulting in a slow standalone heating rate. However, when the Ni substrate is immersed in the solution, the strong contrast in conductivity and permittivity at the interface leads to interfacial charge accumulation. This, combined with the geometric field enhancement provided by the porous structure of the Ni foam, intensely concentrates the electric field lines at the substrate surface. The power density (*P*) dissipated as heat by the dielectric solution is governed by the relationship,

$$P\;=\;2\;\pi \;f\;\varepsilon 0\;\varepsilon \prime \prime \;|E{|}^{2}$$

where *P* is proportional to the square of the electric field (|*E*|^2^). Consequently, the localized amplification of the field at the Ni interface, coupled with the substantial ε″ of the solution, triggers an exponential surge in energy absorption. This energy concentration induces interfacial superheating, creating a high‐temperature reaction zone at the substrate surface that facilitates an ideal environment for rapid and efficient catalyst synthesis.

These simulation and experimental results elucidate that the specific mode of localized heating is critical for establishing a highly favorable environment for Fe‐Ni_3_S_2_ catalyst synthesis. While field enhancement in the foil is confined to macroscopic geometric singularities, the 3D mesh architecture of the Ni foam facilitates a dense network of microwave hotspots across its entire volume. The fundamental difference lies in the spatial density of field‐enhancing sites. In the Ni foil, MW field enhancement is confined only to macroscopic geometric singularities, such as sharp edges and corners, leading to sporadic and localized “hot zones.” In contrast, the 3D porous architecture of the Ni foam consists of an interconnected mesh of ligaments and junctions that act as a dense network of microscopic field concentrators throughout the entire volume of the support. This 3D network influences the catalyst formation process in two critical ways: it brings uniformity of reaction sites, and it can also accelerate reaction kinetics. Unlike the foil, where growth is restricted to the edges, the pervasive hotspots in the Ni foam ensure that the thermal energy required for precursor decomposition is available across the entire surface area, leading to comprehensive catalyst coverage. According to the Arrhenius law (*k* = *A* exp(‐*E*_a_/*RT*)), the intense localized superheating at these junctions significantly lowers the kinetic barrier for reaction. This promotes rapid precursor decomposition and enhances ionic mobility toward the substrate [40], facilitating the growth of high‐quality crystals with superior structural integrity within a minimal timeframe.

### 2.2 Optimizing the Conditions for the High‐Quality Catalyst Synthesis

We investigated the catalyst formation by using field‐emission scanning electron microscopy (FE‐SEM) to confirm the synthesis of the Fe‐Ni_3_S_2_. For the synthesis of a uniform and highly crystalline Fe‐Ni_3_S_2_ on the Ni foam, we optimized the concentrations of the sulfur source and thiourea in the precursor solution. Starting with a solution of Ni(NO_3_)_2_ · 6H_2_O (30 mM) and Fe(NO_3_)_3_·9H_2_O (10 mM) in 2 mL of deionized (DI) water, we compared thiourea concentrations of 360, 720, and 1080 mM. A too low concentration (360 mM) of the precursor solution resulted in the formation of a catalyst with poor crystallinity, whereas 1080 mM led to the presence of surface byproducts. The optimal condition was 720 mM for all syntheses (Figure S3), causing no byproducts and producing high‐quality crystals. Next, we optimized the MW power by testing 50, 75, and 100 W for a duration of 20 min. Figure S4 exhibits the MW time dependence of the crystal growth. Full growth of high‐quality crystals required more than 12 min. Applying 75 W for 20 min produced sharp and high‐crystallinity structures among the three MW powers (Figure S5). Notably, through time‐dependent experiments, we confirmed that both the morphology and electrochemical properties reached a saturation point at 15 min. As shown in Figure S6, extending the reaction duration to 20 min resulted in no significant variations in the structural features or OER performance. Consequently, 15 min was established as the standard reaction time for all subsequent characterizations and evaluations to highlight the exceptional process efficiency of our microwave‐assisted synthesis. The final optimization step was taking control over the coverage of the metal support by the Fe‐Ni_3_S_2_ catalyst. We adjusted the catalyst loading by preparing the precursor solutions with a varied molar concentration. The optimization conditions for the synthesis of a uniform high‐quality Fe‐Ni_3_S_2_ catalyst were as follows: a precursor solution of 30 mM of Ni(NO_3_)_2_ · 6H_2_O, 10 mM of Fe(NO_3_)_3_·9H_2_O, and 720 mM of thiourea, treated for 20 min under 75 W MW power. Figure S7 shows that half of this optimized concentration resulted in insufficient coverage by the Fe‐Ni_3_S_2_, whereas twice the optimized concentration caused an excessively thick film that was prone to delamination. It is noteworthy that using Ni foil as a substrate led to a high concentration of Fe‐Ni_3_S_2_ at the edges of the foil, as predicted by the simulation. This edge‐focused growth produced a bulky layer with extensive cracks (Figure S8), likely due to nonuniform crystal growth.

### 2.3 Structural and Morphological Characterization of the Catalyst on the Ni Foam

**FIGURE 3:**
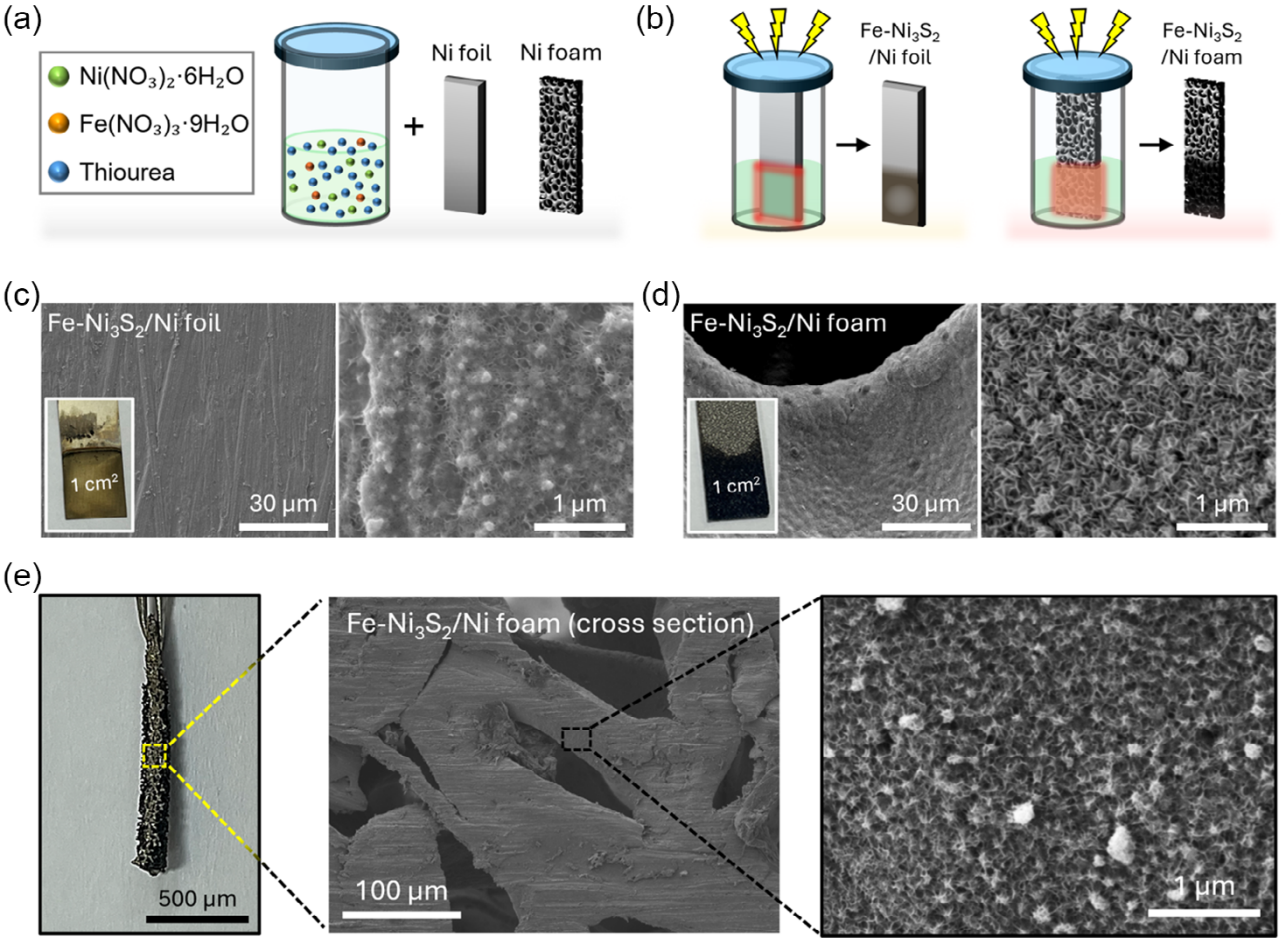
Synthesis and morphological characterization of Fe‐Ni_3_S_2_. (a,b) Schematic diagram illustrates the microwave synthesis of Fe‐Ni_3_S_2_, from the (a) initial precursors solution and substrates to the (b) final products. SEM images of the Fe‐Ni_3_S_2_ synthesized on (c) Ni foil and (d) Ni foam. (e) Cross‐sectional optical image and SEM image of the Fe‐Ni_3_S_2_/Ni foam.

We synthesized the Fe‐Ni_3_S_2_ catalyst on the Ni foil and the Ni foam by using the optimal conditions to investigate the dependence of the substrate. The Ni foil and Ni foam substrates were immersed in the precursor solution contained in a vial (5 mL), and MW (75 W) was applied to the vial for 15 min, resulting in the formation of Fe‐Ni_3_S_2_/Ni foil and Fe‐Ni_3_S_2_/Ni foam (Figure 3a,b). The insets of Figure 3c,d exhibit camera images of the products. The Ni foil caused an exclusively large concentration of the Fe‐Ni_3_S_2_ at the edges of the foil. The Fe‐Ni_3_S_2_ nanostructures on these edges produced a thick, bulky layer with extensive cracks. The central area of the Ni foil was covered by a thinner, rough Fe‐Ni_3_S_2_ layer composed of small, aggregated particles with poorly defined features, suggesting less‐developed crystal growth (Figure 3c). In contrast, the formation of the Fe‐Ni_3_S_2_ catalyst on the Ni foam was uniform in the entire area (Figure 3d). Furthermore, inspecting the cross‐section SEM images (Figure 3e) prepared by cutting the foam confirmed that the catalyst crystals were uniformly created not only on the outer surfaces but also on the entire 3D architecture, which is due to the MW field enhancement and volumetric heating predicted by the simulation.

**FIGURE 4:**
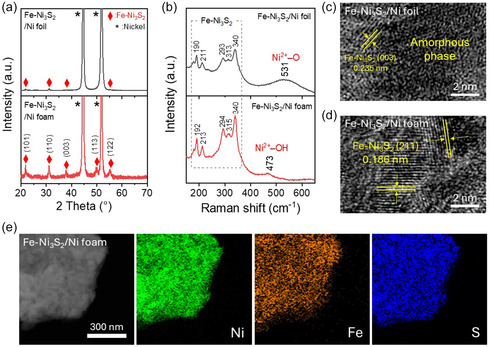
Structural/compositional characterization of Fe‐Ni_3_S_2_ grown on Ni foil and Ni foam: (a) XRD patterns and (b) Raman spectra. HR‐TEM image of (c) Fe‐Ni_3_S_2_/Ni foil and (d) Fe‐Ni_3_S_2_/Ni foam. (d) EDS mapping analysis of the Fe‐Ni_3_S_2_/Ni foam.

The X‐ray diffraction (XRD) patterns of both Fe‐Ni_3_S_2_/Ni foil and Fe‐Ni_3_S_2_/Ni foam were in good agreement with the standard XRD data of pure Ni_3_S_2_ (JCPDS No. 44–1418) (Figure 4a), with the main peaks of 22.1°, 31.5°, 38.1°, 50.0°, and 55.5°, which correspond to (101), (110), (003), (113), and (122), respectively [17]. The two intense peaks at 44.5° and 51.8° are (111) and (200) of the Ni substrate (JCPDS No. 04–0850) [22]. The Fe‐Ni_3_S_2_/Ni foam exhibited a higher crystallinity than that of Fe‐Ni_3_S_2_/Ni foil, consistent with SEM observations. The Raman spectra for both samples confirmed the presence of the Fe‐Ni_3_S_2_ phase (Figure 4b). Characteristic peaks of the Fe‐Ni_3_S_2_/Ni foam were observed at 192, 213, 294, 315, and 340 cm^−1^ [28]. In addition, the Fe‐Ni_3_S_2_/Ni foam exhibited a strong, distinct peak at ∼472 cm^−1^, which is one of the primary vibrational modes for the NiOOH active phase. Dominance of the band, typically found near 475 and 555 cm^−1^, is well‐known to host the catalytically active sites for the OER [41]. Although the Fe‐Ni_3_S_2_/Ni foil exhibited similar Raman peaks at approximately the same positions, it showed a peak at 531 cm^−1^, which indicates the presence of amorphous or defect‐rich states of the Fe‐Ni_3_S_2_. Such rich defects are reported to be detrimental to catalytic activity [42].

To investigate the nanostructure and elemental composition through transmission electron microscopy (TEM), the Fe‐Ni_3_S_2_ crystals were detached from the substrate via high‐power ultrasonication. A large fraction of the Fe‐Ni_3_S_2_ foil exhibited a disordered atomic arrangement with no clear lattice fringes (Figure 4c), despite the localized presence of crystalline domains with a spacing of 0.238 nm corresponding to the (003) plane of Fe‐Ni_3_S_2_ [43]. Furthermore, energy‐dispersive X‐ray spectroscopy (EDS) mapping revealed a clear spatial segregation of the elements (Figure S9, Table S2). These findings suggest the presence of predominantly amorphous regions and uneven decomposition of the precursor. In contrast, a low‐magnification scanning transmission electron microscopy (STEM) image of the Fe‐Ni_3_S_2_/Ni foam showed nanoclustering of the crystals (Figure S10), and the high‐resolution TEM (HR‐TEM) image (Figure 4d) exhibited distinct lattice fringes with a spacing of 0.186 nm corresponding to the (211) plane of Ni_3_S_2_ [44]. EDS mapping revealed a homogeneous distribution of Ni, Fe, and S elements, along with a minor presence of oxygen (Figure 4e, Figure S11), which confirmed uniform doping of Fe in Ni_3_S_2_. The atomic fraction of Fe doped in Ni_3_S_2_ was approximately 10.3 at% (Table S2).

The morphological and structural differences observed in Figures 3 & 4 are a consequence of the substrate‐dependent MW heating characteristics. Specifically, the Fe‐Ni_3_S_2_ synthesized on the Ni foam exhibits significantly higher crystallinity and a more homogeneous surface morphology compared to the less‐ordered structures found on the Ni foil. This is because the formation of a high‐quality metal sulfide phase requires sufficient thermal energy to facilitate rapid nucleation and subsequent crystal growth. While the limited heating on the Ni foil fails to provide a consistent thermal landscape, the 3D interconnected mesh of the Ni foam generates a high‐density network of interfacial hotspots. According to the relationship between synthesis temperature and crystallinity in metal sulfides, a higher thermal input during the reaction promotes the arrangement of atoms into a well‐defined lattice, thereby reducing amorphous defects and enhancing structural integrity. Consequently, the intense and uniform surface heating enabled by the Ni foam architecture provides the ideal thermodynamic environment for the exclusive growth of high‐performance, high‐crystallinity Fe‐Ni_3_S_2_ catalysts across the entire electrode surface.

### 2.4 Electrocatalytic OER Performance of the Catalysts

The electrocatalytic OER performance of the Fe‐Ni_3_S/Ni foil was evaluated in a three‐electrode system using Ar‐purged 1 M KOH electrolyte at room temperature. An Hg/HgO (1M NaOH) reference electrode was calibrated by measuring the H_2_ oxidation/evolution on a Pt rotating disk electrode, and all electrode potentials were converted to the reversible hydrogen electrode (RHE) [45]. The OER polarization curves were collected by cyclic voltammetry (CV). Prior to measurement, each electrode was activated by a constant‐potential protocol until the OER current was stabilized (Figure S12), after which the final CV scan was analyzed [46].

**FIGURE 5:**
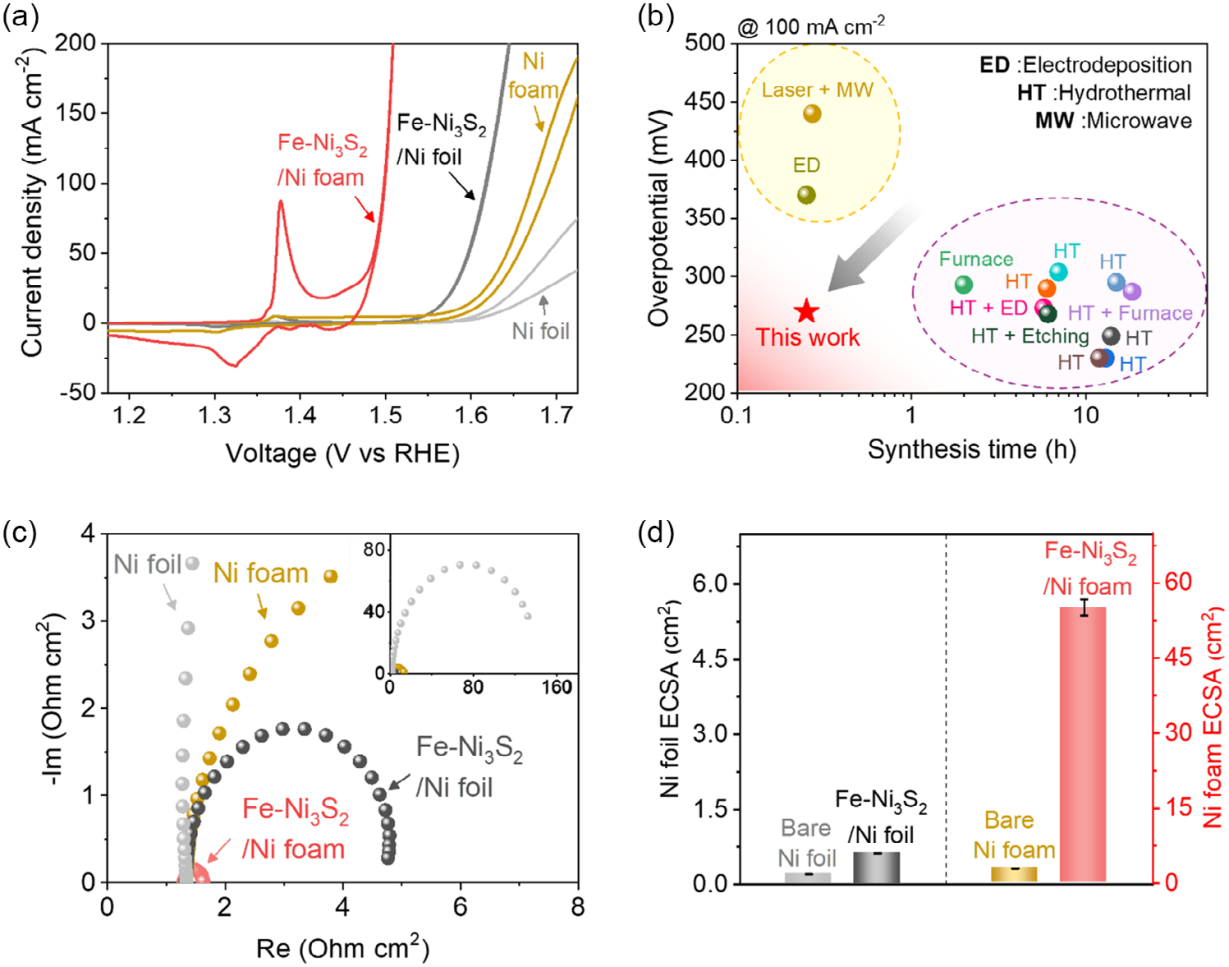
Electrochemical performances of bare Ni foil and Ni foam, Fe‐Ni_3_S_2_/Ni foil and Fe‐Ni_3_S_2_/Ni foam: (a) The OER polarization curve. (b) Synthesis time and OER performance comparison with previously reported NiFe‐based OER catalysts synthesized via various methods. (c) Nyquist plots at an overpotential of 300 mV. (d) Comparison of the electrochemically active surface area (ECSA).

The Fe‐Ni_3_S_2_/Ni foil required a high overpotential of *η* = 0.387 V at 100 mA cm^−2^, even though its performance was better than that of a bare Ni foil. The Fe‐Ni_3_S_2_/Ni foam exhibited markedly superior OER activity, achieving an overpotential of *η* = 0.268 V at 100 mA cm^−2^ (Figure 5a), which is remarkably low among a wide range of NiFe‐based OER electrocatalysts prepared by diverse synthetic routes (Figure 5b and Table S3). In order to achieve comparable performance, majority of recently reported electrocatalysts typically require significantly long synthesis durations. For instance, conventional high‐temperature synthesis routes such as hydrothermal methods often demand extensive reaction times, ranging from 6 h to over 18 h [17, 22, 23, 24, 25, 26, 27, 28], and the processes involving furnace annealing typically require 2 h of treatment [4, 47]. Rapid synthesis offers a significant time advantage; however, many of such methods compromise catalytic performance. For example, techniques like electrodeposition (∼15 min), which often face limitations in precise compositional control, and laser‐assisted synthesis (∼16 min), despite being fast, result in catalysts with high overpotentials of 370 and 440 mV at 100 mA cm^−2^, respectively [29, 30, 31]. The rapid MW‐assisted synthesis (15 min) developed in this study not only matches the rapid synthesis but also achieves a much lower overpotential of 270 mV at 100 mA cm^−2^.

To further compare the OER kinetics, electrochemical impedance spectroscopy (EIS) was conducted at *η* = 0.300 V. In Nyquist plots (Figure 5c), the charge transfer resistance (R_ct_), represented by the semicircle diameters, had the following trend: Fe‐Ni_3_S_2_/Ni foam (0.3 Ω cm^−2^) < Fe‐Ni_3_S_2_/Ni foil (3.4 Ω cm^−2^) < bare Ni foam (13 Ω cm^−2^) << bare Ni foil (150 Ω cm^−2^). Consistently, the Fe‐Ni_3_S_2_/Ni foam showed the smallest Tafel slope among all samples (Figure S13), in agreement with its highest OER activity. The superior OER activity of Fe‐Ni_3_S_2_/Ni foam can be explained by uniform crystal growth across the substrate. We further estimated the electrochemically active surface area (ECSA) from double‐layer capacitance obtained in CV (Figure S14). The ECSA of Fe‐Ni_3_S_2_/Ni foil was 0.63 cm^2^, which was only three times higher than that of bare Ni foil (0.21 cm^2^, Figure 5d). In contrast, the ECSA of Fe‐Ni_3_S_2_/Ni foam reached 55.13 cm^2^, which was 18.4 times higher than that of bare Ni foam (3 cm^2^). The substantially enlarged ECSA of the Fe‐Ni_3_S_2_/Ni foam reduces the effective current density per active site and promotes more efficient interfacial charge transport. Consequently, the reduced R_ct_ directly contributes to the lower overpotential observed at high current densities, confirming a clear structure–property–activity relationship.

### 2.5 Surface States of the Catalysts during OER

**FIGURE 6:**
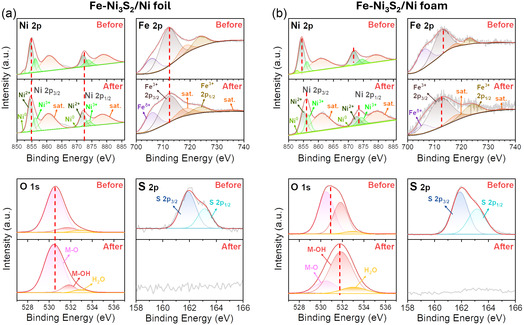
XPS investigation of the surface chemical state changes during the OER process. Comparison of Ni 2p, Fe 2p, O 1s, and S 2p XPS spectra for (a) Fe‐Ni_3_S_2_/Ni foil and (b) Fe‐Ni_3_S_2_/Ni foam before and after OER.

To investigate the differences in catalytic activity of Fe‐Ni_3_S_2_ synthesized on different substrates, we performed an analysis of the surface states of the catalysts for the as‐prepared and post‐OER samples by using X‐ray Photoelectron Spectroscopy (XPS). The Fe‐Ni_3_S/Ni foil exhibited considerable surface inertness during the OER (Figure 6a). The Ni 2p spectra before and after the reaction remained largely unchanged, as evidenced by the absence of any significant peak shift in the Ni 2p_3/2_ (854.7 eV) and Ni 2p_1/2_ (872.2 eV) [48]. This suggests that the oxidation of Ni, a necessary step for OER, was suppressed. After the reaction, the relative intensity of the initial Fe^3+^ peak decreased, while the contribution from lower oxidation states, such as Fe^2+^ (denoted as δ^+^), increased. This paradoxical reduction of Fe is considered to take place because the typical pathway for Ni and Fe oxidation and reconstruction into (oxy)hydroxides was inhibited on the Ni foil surface. This phenomenon suggests that a parasitic reaction, the oxidation of sulfide (S^2−^) to sulfate (SO_4_^2−^), occurs concurrently, releasing electrons that accumulate on the passivated surface to create a micro‐reductive environment, which in turn reduces adjacent Fe^3+^ ions to Fe^2+^ [49, 50]. This mechanism is supported by the S 2p spectra, where the initial sulfide peaks (S 2p_3/2_ and S 2p_1/2_) completely disappear after the OER, indicating the complete removal or oxidation of surface sulfur. Additionally, the O 1s spectra after the OER showed increased intensities in the metal‐hydroxide (M‐OH) and adsorbed water (H_2_O) components, but no significant structural changes for active phase formation were observed.

In contrast, the Fe‐Ni_3_S_2_/Ni foam underwent a significant surface transformation during OER (Figure 6b). The post‐OER Ni 2p spectrum for the Ni foam revealed a significant shift of the main peaks to higher binding energies; the Ni 2p_3/2_ peak shifted to 856.1 eV and the Ni 2p_1/2_ peak to 873.9 eV, compared to their initial positions before the OER (854.7 and 872.6 eV, respectively) [51, 52]. This positive shift in binding energy is a direct consequence of the markedly increased intensity of the Ni^3+^ species, which confirmed the successful oxidation of Ni^2+^ to a higher, catalytically active valence state [53]. Likewise, the Fe 2p spectrum showed a significant increase in the proportion of Fe^3+^, indicating that iron was also oxidized under OER conditions [52]. This comprehensive surface reconstruction is further corroborated by the O 1s and S 2p spectra. The O 1s spectrum after OER exhibits a substantial increase in the metal‐hydroxide (M‐OH) peak, providing direct evidence for the formation of (oxy)hydroxide species [52, 54]. Concurrently, the complete disappearance of the sulfide peaks in the S 2p spectrum indicates that the initial sulfide precatalyst surface has been fully converted. The significant increase in the surface oxidation states of both Ni^3+^ and Fe^3+^, combined with the prominent M‐OH signal, provides direct evidence for the in‐situ formation of nickel iron (oxy)hydroxide (Ni, FeOOH), which is the active species for the OER [55].

Further analysis by Raman spectroscopy revealed critical differences in the states of the catalysts after the CV scans (Figure S15). For the Fe‐Ni_3_S_2_/Ni foil, a broad and weak NiOOH peak was observed at 519 cm^−1^, which is likely a convolution of the characteristic NiOOH bands at 474 and 554 cm^−1^ [17]. In contrast, the Fe‐Ni_3_S_2_/Ni foam showed distinct FeOOH peaks at 235 and 359 cm^−1^, along with a broad and strong NiOOH peak at 543 cm^−1^ [28]. These Raman findings, combined with the XPS surface analysis, confirm that the Fe‐Ni_3_S_2_/Ni foam acted as a highly effective precatalyst, transforming into this active oxyhydroxide phase during the reaction to deliver its outstanding performance.

These fundamental differences in surface evolution are closely related to the substrate‐dependent microwave absorption and the resulting crystallinity of the catalysts. The porous structure of the Ni foam enables more uniform and intense microwave absorption compared to the Ni foil, leading to the formation of Fe‐Ni_3_S_2_ with superior crystallinity. During the OER activation process, this high‐quality crystalline phase on the foam substrate promotes a more efficient and thorough surface reconstruction into the active Ni(Fe)OOH phase. In contrast, the relatively lower crystallinity and limited microwave absorption on the Ni foil result in surface inertness and the suppression of the necessary Ni oxidation to higher valence states. Consequently, the foam‐based catalyst provides a much higher density of active oxyhydroxide sites, which is the primary factor for its outstanding OER performance.

### 2.6 OER Durability Test and Postcatalytic Analysis of the Fe‐Ni_3_S_2_/Ni Foam

**FIGURE 7:**
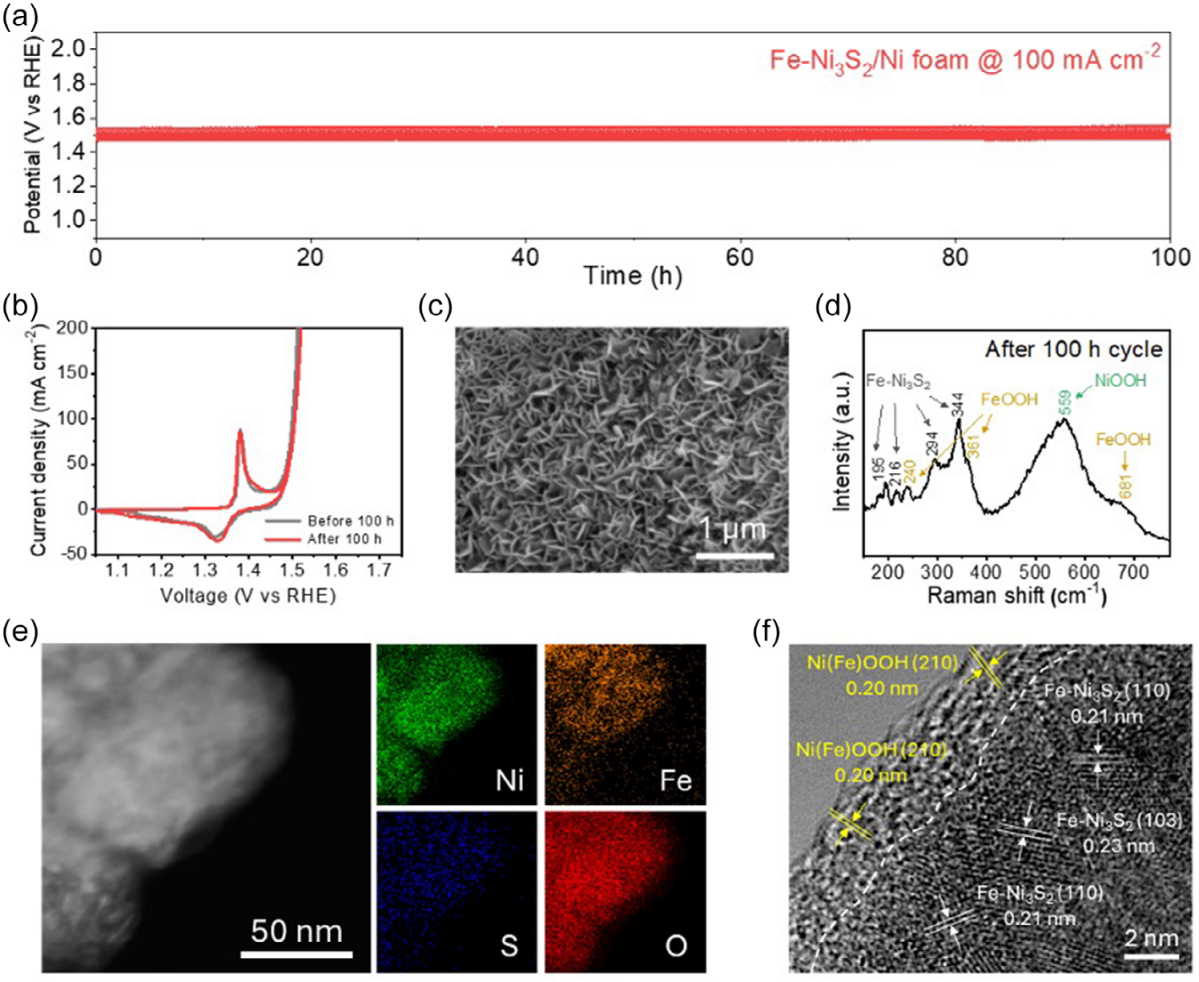
Long‐term stability evaluation of the Fe‐Ni_3_S_2_ catalyst. (a) Chronopotentiometry test at a constant current density (100 mA cm^−2^) for 100 h. (b) CV curves recorded before and after long‐term cycle. Characterization of the catalyst after long‐term cycle: (c) SEM image, (d) Raman spectrum, (e) TEM image and corresponding EDS elemental mapping, and (f) HR‐TEM image.

The durability of the catalyst is a crucial requirement for advanced electrocatalysts. To evaluate its durability, we performed a chronopotentiometry (CP) test at a current density of 100 mA cm^−2^ for the Fe‐Ni_3_S_2_/Ni foam. The catalyst maintained a stable potential for 100 h with negligible degradation (Figure 7a), and the posttest CV curve showed only a minimal shift of 4 mV at a current density of 100 mA cm^−2^ relative to the initial one (Figure 7b). Detailed structural and compositional analyses after the durability test provided comprehensive evidence for the outstanding stability of the catalyst. The SEM image in Figure 7c reveals that the initial nanosheet morphology was well‐preserved after 100 h of continuous operation, indicating its excellent structural robustness. Raman spectroscopy was conducted to probe the surface changes after the long‐term cycles (Figure 7d). Compared to the state after initial CV scan activation, the spectrum shows a significant increase in the intensity of the peaks related to NiOOH and FeOOH, while the characteristic peaks of the underlying Fe‐Ni_3_S_2_ core were well‐maintained. Notably, the appearance of a distinct peak around 681 cm^−1^, corresponding to FeOOH, further indicates that both nickel and iron contribute to forming these catalytically active species [56]. This surface reconstruction was further investigated by XPS analysis (Figure S16). The Ni 2p and Fe 2p spectra show the presence of higher oxidation states (Ni^3+^ and Fe^3+^). Notably, the S 2p signal was significantly attenuated, suggesting the formation of a surface layer that covered the initial sulfide. The O 1s spectrum was dominated by metal‐hydroxide (M‐OH) bonds, all of which are consistent with the formation of Ni, FeOOH.

After the 100‐h cycles, the Fe‐Ni_3_S_2_ was detached from the Ni foam for TEM analysis. The EDS mapping of these detached nanoclusters (Figure 7e) reveals that the catalyst was significantly enriched with oxygen, confirming a substantial conversion from the initial sulfide to oxygen‐containing species. More conclusively, the HR‐TEM image (Figure 7f) provides direct evidence of core–shell structure formation. Crystalline lattice fringes belonging to the original Fe‐Ni_3_S_2_ phase are clearly observed in the interior region, with spacings of 0.23 and 0.21 nm corresponding to the (103) and (110) planes, respectively. In addition, the surface region exhibits newly formed domains with a lattice spacing of 0.20 nm, which is assigned to the (210) plane of the Ni(Fe)OOH phase. These results indicate that the conductive Fe‐Ni_3_S_2_ core remains structurally intact while being encapsulated by a catalytically active Ni(Fe)OOH shell [57, 58]. This result indicates that the conductive Fe‐Ni_3_S_2_ core remained intact while being covered by a catalytically active Ni and FeOOH, which is the key to its exceptional long‐term durability [59]. It is important to ensure that this reconstruction is stable and does not lead to element leaching. To quantitatively compare the stability relative to activity, we evaluated the activity‐stability factor (ASF) [60, 61] and the stability number (S‐number) [62]. Both metrics were determined by inductively coupled plasma mass spectrometry (ICP‐MS) measurement of the electrolyte sampled during CP operation at 100 mA cm^−2^. After 100 h, the dissolved Fe and Ni concentrations were 0.629 and 1.774 ppb, respectively. These values correspond to an ASF of 3.37 × 10^7^ and an S‐number of 1.91 × 10^7^ for Fe‐Ni_3_S_2_/Ni foam, highlighting its high stability under OER conditions.

These comprehensive results demonstrate that the exceptional long‐term stability and activity of the catalyst originate from its unique hierarchical configuration. The uniform and intensive microwave absorption facilitated by the foam substrate promotes the formation of a highly crystalline Fe‐Ni_3_S_2_ phase, which serves as a robust and conductive backbone. Even after extended electrochemical cycles, this crystalline core remains structurally intact, while the in‐situ formed Ni(Fe)OOH shell provides a stable environment for continuous catalysis. This self‐reconstructed oxyhydroxide layer acts as a protective barrier that prevents excessive metal leaching, as evidenced by the high values of ASF and S‐number. Consequently, the synergistic combination of a stable crystalline core and a durably reconstructed active shell ensures that the Fe‐Ni_3_S_2_/Ni foam maintains its superior performance throughout long‐term operations without significant structural degradation.

### 2.7 Full‐Cell Performance Evaluation: Alkaline Water Electrolyzer (AWE)

**FIGURE 8:**
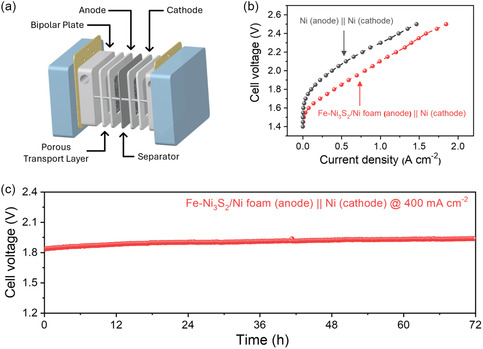
Performance evaluation of the full‐cell alkaline water electrolyzer (AWE). (a) Schematic of the AWE cell configuration. (b) Polarization curves comparing the full cell assembled with the Fe‐Ni_3_S_2_/Ni anode and a standard Ni anode. (c) Long‐term stability test conducted at a constant current density (400 mA cm^−2^).

Guided by the three‐electrode results, we conducted a single‐cell test by using a zero‐gap type AWE. The cell comprised a Zirfon separator, Fe‐Ni_3_S_2_/Ni foam or bare Ni foam anode, a bare Ni foam cathode, Ni foam porous transport layers, Ni end plates, and Au‐coated stainless steel current collectors (Figure 8a). As shown in Figure 8b, the Fe‐Ni_3_S_2_/Ni foam(anode) || bare Ni foam(cathode) cell achieved a current density of 0.80 A cm^–2^ at an applied voltage of 2.0 V, which is 2.1 times that of bare Ni(anode) || bare Ni(cathode) (0.375 A cm^–2^ @ 2.0 V). Notably, this improved full‐cell polarization behavior confirms that the superior intrinsic OER activity identified in the three‐electrode measurements is effectively translated to the device level under practical zero‐gap AWE operation. At an industrially relevant current density of 0.4 A cm^–2^, the Fe‐Ni_3_S_2_/Ni foam anode sustained 72 h of stable full‐cell operation with minimal voltage rise (Figure 8c). Taken together, these results highlight substantial gains in both activity and stability of the Fe‐Ni_3_S_2_/Ni foam catalyst and support its suitability for AWE applications.

## 3 Conclusion

We have developed a rapid and energy‐efficient strategy for the direct synthesis of Fe‐Ni_3_S_2_ catalysts on Ni foam substrate, enabled by MW field enhancement. We elucidated the underlying mechanism, demonstrating that the immersion of the metallic foam substrate in the precursor solution creates a localized enhancement of the electromagnetic field. Specifically, this interfacial microwave enhancement, unique to the 3D metallic framework, enables precise thermal control that ensures the formation of a highly crystalline and conformal coating across the entire substrate. The accelerated MW absorption and heating lead to uniform decomposition of the precursors and conformal coating of the Fe‐Ni_3_S_2_ catalysts on the entire 3D network Ni foam substrate. The rapid synthesis (15 min) at a low MW power (75 W), compared to conventional synthesis methods, is advantageous for scale‐up production of the catalyst. The resulting Fe‐Ni_3_S_2_/Ni foam, possessing excellent crystallinity and a uniform morphology, exhibited outstanding OER electrochemical performance, delivering a current density of 100 mA cm^−2^ at a low overpotential of 270 mV and exceptional long‐term stability for over 100 h. This excellent performance is attributed to its effective in‐situ transformation into the highly active Ni and FeOOH phases. Furthermore, its successful implementation as an anode in a real AWE cell highlights its viability in industrial applications. Collectively, these findings establish that employing MW field enhancement is a powerful and versatile strategy, paving the way for the future design of advanced OER electrocatalysts.

## 4 Experimental Section

### 4.1 Preparation of Fe‐Ni3S2 on Electrodes

The nickel foil (35 × 10 × 0.3 mm, MTI Co. Ltd., Korea) and foam (35 × 10 × 1.6 mm, MTI Co. Ltd., Korea) substrates were ultrasonically cleaned in acetone (Samchun Pure Chemical Co. Ltd., Korea), 3.0 M hydrochloric acid (Samchun Pure Chemical Co. Ltd., Korea), deionized (DI) water, and ethanol (Samchun Pure Chemical Co. Ltd., Korea) each for 10 min to remove surface contaminants such as oils and oxides. The cleaned nickel foil and foam were rinsed with DI water several times and dried in a vacuum oven at 60°C for 3 h. To prepare the precursor solution, 17.44 mg of nickel(II) nitrate hexahydrate (Ni(NO_3_)_2_ · 6H_2_O, 30 mM, Sigma–Aldrich), 8.08 mg of iron(III) nitrate nonahydrate (Fe(NO_3_)_3_·9H_2_O, 10 mM, Sigma–Aldrich), and 109.6 mg of thiourea (720 mM, Sigma–Aldrich) were dissolved in 2 mL of DI water to form a homogeneous precursor solution. Subsequently, a piece of the precleaned substrate (nickel foil or nickel foam) was immersed in the precursor solution. The synthesis was then carried out via microwave irradiation at a power of 75 W for 15 min. After the reaction, the as‐prepared sample was thoroughly rinsed several times with DI water and ethanol to remove any unreacted precursors. Finally, the catalyst was dried in a vacuum oven at 60°C for 6 h.

### 4.2 Characterizations

The morphology of the samples was analyzed using a field‐emission scanning electron microscope (FE‐SEM, JSM 7800F PRIME, JEOL.LTD). The crystallographic structures were investigated by XRD using a RIGAKU D/MAX‐2500/PC diffractometer. Raman spectra were acquired on a micro‐Raman spectrometer (Witec Alpha 300 RA Confocal Raman) with a 532 nm laser excitation and a spot size of 1 μm. For detailed microstructural analysis, transmission electron microscopy (TEM, JEM‐2100) and high‐angle annular dark‐field scanning transmission electron microscopy (HAADF–STEM, Cs‐corrected JEM‐2100F STEM) were employed, both operating at an accelerating voltage of 200 kV. The chemical composition and electronic states of the samples were determined by X‐ray photoelectron spectroscopy (XPS, VG SCIENTIFIC ESCALAB 250). Additionally, the concentration of dissolved Ni and Fe released during the OER was quantified by inductively coupled plasma mass spectrometry (ICP‐MS, NexION 300S, PerkinElmer), for which concentrations were obtained from linear‐fitted calibration curves (1, 5, and 10 ppb) after diluting the samples to fit within the calibration range.

## Supporting Information

Additional supporting information can be found online in the Supporting Information section. **Supporting Fig. S1:** Configuration of a custom‐built single‐mode MW reactor. **Supporting Fig. S2:** Simulated dielectric loss distribution of (a) plate structure and (b) mesh structure. **Supporting Fig. S3:** Morphology of the Fe‐Ni_3_S_2_ catalyst synthesized on Ni foam with varying thiourea concentrations in a precursor solution of 30 mM Ni(NO_3_)_2_ · 6H_2_O and 10 mM Fe(NO_3_)_3_·9H_2_O. **Supporting Fig. S4:** Time‐dependent ex‐situ SEM images showing the morphological evolution of Fe‐Ni_3_S_2_ on a Ni foam. **Supporting Fig. S5:** Morphology of the Fe‐Ni_3_S_2_ catalyst synthesized on Ni foam with varying microwave power with a precursor solution of 30 mM Ni(NO_3_)_2_ · 6H_2_O, 10 mM Fe(NO_3_)_3_·9H_2_O and 720 mM thiourea. **Supporting Fig. S6:** Structural and electrochemical characterization of the Fe‐Ni_3_S_2_/Ni foam synthesized for 20 min. **Supporting Fig. S7:** Morphology and crystallinity of the Fe‐Ni_3_S_2_ catalyst synthesized on Ni foam using solutions at half and double the concentration of the optimized precursor solution. **Supporting Fig. S8:** A low‐magnification FE‐SEM image of the Fe‐Ni_3_S_2_ catalyst synthesized on a Ni foam substrate under optimized conditions. **Supporting Fig. S9:** EDS elemental maps of Fe‐Ni_3_S_2_ synthesized on Ni foil. **Supporting Fig. S10:** Low‐magnification STEM image of Fe‐Ni_3_S_2_ on Ni foam. **Supporting Fig. S11:** EDS oxygen maps of Fe‐Ni_3_S_2_ synthesized on Ni foam. **Supporting Fig. S12:** Catalytic activation of the Ni foil and foam, Fe‐Ni_3_S_2_/Ni foil and Fe‐Ni_3_S_2_/Ni foam by cyclic voltammetry. **Supporting Fig. S13:** Tafel plots for the OER on Ni foil and foam, Fe‐Ni_3_S_2_/Ni foil and Fe‐Ni_3_S_2_/Ni foam. **Supporting Fig. S14:** Double‐layer capacitance (C_dl_) for estimating the electrochemically active surface area in 1 M KOH. (a), (c), (e), (g) Cyclic voltammograms collected within a non‐Faradaic potential window at various scan rates. (b), (d), (f), (h) Cathodic (black circles) and anodic (red circles) charging currents at 0.80 V vs RHE plotted as a function of scan rate. C_dl_ was determined as the average of the absolute values of the slopes obtained from linear fits to the cathodic and anodic datasets. **Supporting Fig. S15:** Raman spectroscopy of Fe‐Ni_3_S_2_/Ni foil and Fe‐Ni_3_S_2_/Ni foam after OER. **Supporting Fig. S16:** XPS spectra of the Ni 2p, Fe 2p, S 2p, and O 1s for Fe‐Ni_3_S_2_/Ni foam after the 100 h durability test. **Supporting Table S1:** Dielectric property of a precursor solution containing 30 mM Ni(NO_3_)_2_ · 6H_2_O, 10 mM Fe(NO_3_)_3_·9H_2_O, and 720 mM thiourea dissolved in deionized (DI) water. **Supporting Table S2:** Comparison of the quantitative elemental analysis for Fe‐Ni_3_S_2_ catalysts synthesized on Ni foam versus Ni foil. **Supporting Table S3:** Comparison of OER overpotential and synthesis time with recently reported Ni, Fe‐based catalysts.

## Funding

This study was supported by National Research Foundation of Korea (RS‐2024−00338686, RS‐2025−02303249) and National Research Council of Science and Technology (25A01043).

## Conflicts of Interest

The authors declare no conflicts of interest.

## Supporting information

Supplementary Material

## Data Availability

The data that support the findings of this study are available from the corresponding author upon reasonable request.
