# Supplementary material for: Microwave Field Enhancement at Metal‐Electrolyte Interfaces Enables Rapid Growth of Fe‐Ni3S2 on Nickel Foam for Alkaline Oxygen Evolution
Source: Small Sci. 2026 Apr 4;6(4):e70267. doi: 10.1002/smsc.70267 (PMC13051805; doi:10.1002/smsc.70267)
Supplement: Supplementary file 1 — Supplementary Material [file SMSC-6-e70267-s001.pdf]

Supporting Information

Microwave Field Enhancement at Metal-Electrolyte Interfaces Enables Rapid Growth of Fe–Ni_3S_2 on Nickel Foam for Alkaline Oxygen Evolution

Dongbeom Kim[1‡], Sang-Mun Jung[1‡], Byung-Jo Lee[1], Sangmin Ryu[1], Wonjoon Choi[1], Yoonsun Choi[1], Geonwoo Kim[1], Ji Hye Kwak[2], Sunshin Jung[2], Yong-Tae Kim[1*], and Unyong Jeong[1*]*

Electrochemical measurements

All electrochemical measurements were performed on an electrochemical workstation (Metrohm Autolab PGSTAT204) in a standard three-electrode cell using Ar-saturated 1.0 M KOH (DAEJUNG, 99.9%). The prepared electrode served as the working electrode, a Hg/HgO (1.0 M NaOH) electrode as the reference, and a graphite rod as the counter. All potentials were converted to the reversible hydrogen electrode (RHE). The Hg/HgO reference was calibrated against RHE in H_2-saturated electrolyte using a Pt disk working electrode.[1]

OER polarization curves were recorded by cyclic voltammetry (CV) at 10 mV s^{-1} with iR_s compensation. The solution resistance (R_s) was obtained by electrochemical impedance spectroscopy (EIS) at open-circuit potential. Prior to data collection of OER polarization curves, each electrodes were activated by a constant-potential protocol (holding at 1.7 V vs. RHE) following the reported procedure until the OER current stabilized, after which the final CV scan was analyzed.[2] Tafel plots were derived by replotting the polarization data as overpotential (η) versus log current density (log j).

EIS for charge transfer resistance (R_{ct}) was performed at 1.53 V vs. RHE with an amplitude of 10 mV sinusoidal wave over 10^5–10^{-1} Hz. A 100 h stability test was conducted, and a post-test polarization curve was subsequently measured to compare the electrocatalytic activity.

Electrochemically active surface areas (ECSAs) of the catalysts were estimated from the electrochemical double-layer capacitance (C_{dl}) obtained by CV in a non-Faradaic window (≈ 0.75 to 0.85 V vs. RHE) at a scan rate ranging from 5 to 400 mV s^{-1}.[3] C_{dl} was taken from the slope of capacitive current density versus scan rate.

Activity-Stability Factor (ASF) & Stability number (S-number) calculation

Activity–stability factor (ASF) was evaluated as reported previously.[4] The equivalent dissolution current density, S (mA cm^{-2}), was obtained from the ICP-MS-measured dissolved metal concentration according to Eq. (1):

$$S = \frac{n \cdot C_d \cdot F \cdot V}{M_w \cdot A} \quad \text{(eq.1)}$$

Where n is the valence of the dissolved species, F is the Faraday coefficient (96,485 C mol^{-1}), C_d is the dissolved metal concentration on a mass basis (ppb), V is the ele ctrolyte volume (mL), M_w is the molar mass (g mol^{-1}), A is the geometric area A of the working electrode (cm^2). The ASF was then calculated using eq. 2:

$$ASF = \frac{J-S}{S} \quad \text{(eq.2)}$$

where J is the current density (mA cm^{-2}).

The S-number was calculated following eq.3 from the previous literature.[5] The S-number could be expressed the number of oxygen molecules(n_{O2}) are formed per metal atom lost into the electrolyte (n_{ion}).

$$S - number = \frac{n_{O2}}{n_{ion}} \quad \text{(eq.3)}$$

Water Splitting Investigation: Alkaline Water Electrolyzer (AWE)

A zero-gap alkaline single cell (2×2 cm^2) was assembled with the prepared OER electrodes as anodes, commercial Ni foam electrodes as cathodes, a Zirfon separator (Agfa), Ni foam porous transport layers, Ni end plates, and Au-coated stainless steel current collectors.[6,7] Electrolyte circulation was provided by a peristaltic pump (WT60 0-1F-1C, LONGER) through PharMed BPT tubing (90 cm in length with an internal diameter of 6.3 mm). Cell performance was evaluated in 30 wt% KOH (DAEJUNG) at 80 °C with a flow rate of 300 mL min^{-1}.

Experimental Setup for Microwave Absorption and Temperature Measurement

The microwave absorption properties and corresponding temperature changes were measured using a custom-designed reactor consisting of a WR-340 rectangular wavegu ide. Microwaves were generated at a fixed frequency of 2.45 GHz by a 1.5 kW solid -state power amplifier (SSPA, KRF Co., Ltd). To quantify the power absorbed by the sample, the incident and reflected powers were monitored in real-time with two pow er sensors (U2000, Agilent Technologies) connected to a high-directivity (60 dB) direc tional coupler. Simultaneously, the surface temperature evolution of the sample during irradiation was continuously recorded using an infrared (IR) optical sensor (MI3-LT, R aytek) mounted on the waveguide's exterior.

Computational Simulation Details

To complement the experimental results, the electromagnetic field distribution and microwave absorption within the material were simulated using CST Studio Suite. In the model, a transverse electromagnetic (TEM) mode plane wave was set to propagate from the top (+y direction) towards the sample. To ensure the simulation accurately reflected the experimental conditions, the dielectric properties of the precursor solution were measured and used as input materials parameters. The measurements were performed using a dielectric measurement system based on the open-ended coaxial probe method, which consists of a network analyzer (E5063A, Keysight) and a coaxial probe kit (N1501A, Keysight). The electric field was polarized along the +x-axis with a defined amplitude of 1 V/m and excited with a Gaussian envelope. The computational domain was discretized into approximately 250,000 hexahedral mesh cells to ensure calculation accuracy.

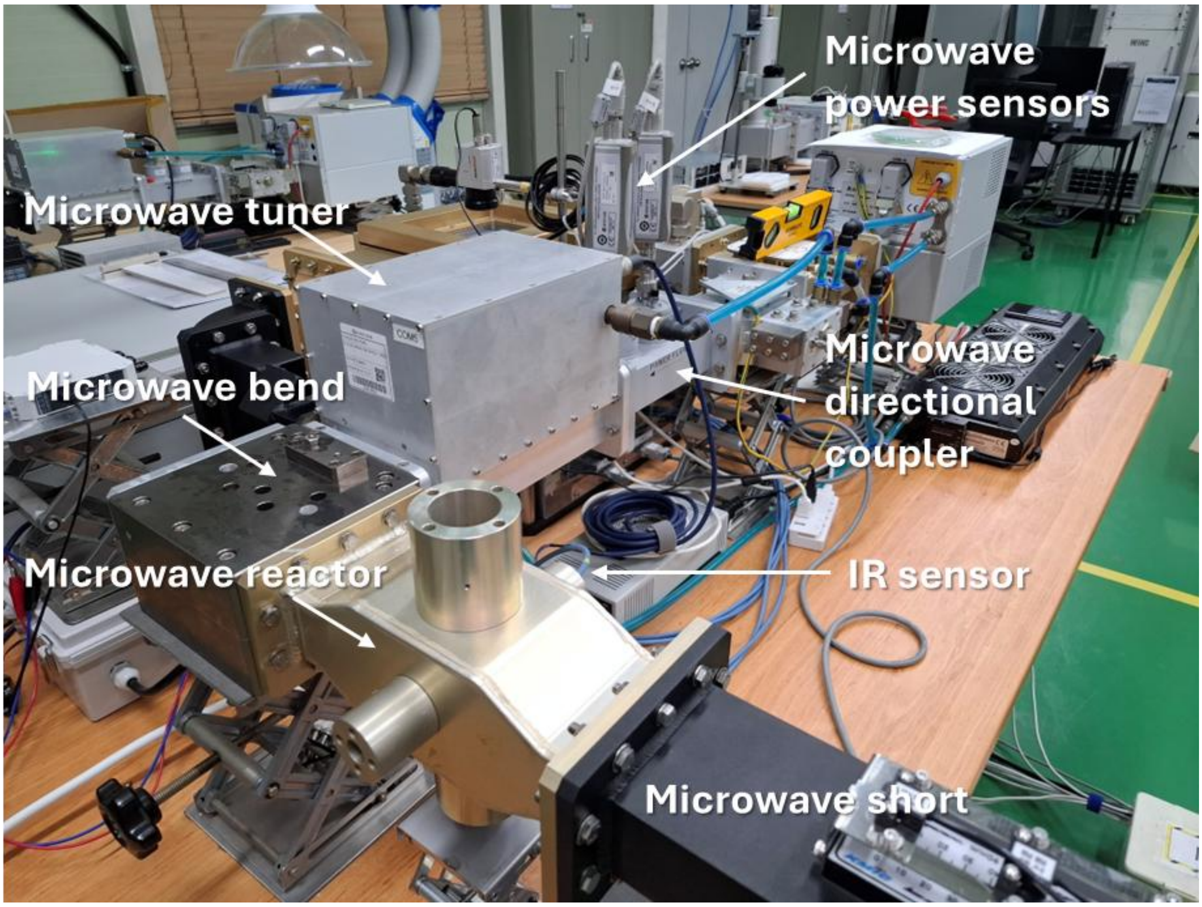

Figure S1. Configuration of a custom-built single-mode MW reactor.

Frequency: 2.45 GHz

	e´ (Real part)	Tand (dielectric loss)
1st	76.5882	0.2093
2nd	76.5800	0.2084
3rd	76.5808	0.2072
Average	76.5830	0.2083

Table S1. Dielectric property of a precursor solution containing 30 mM $Ni(NO_3)_2 \cdot 6H_2O$, 10 mM $Fe(NO_3)_3 \cdot 9H_2O$, and 720 mM thiourea dissolved in deionized (DI) water.

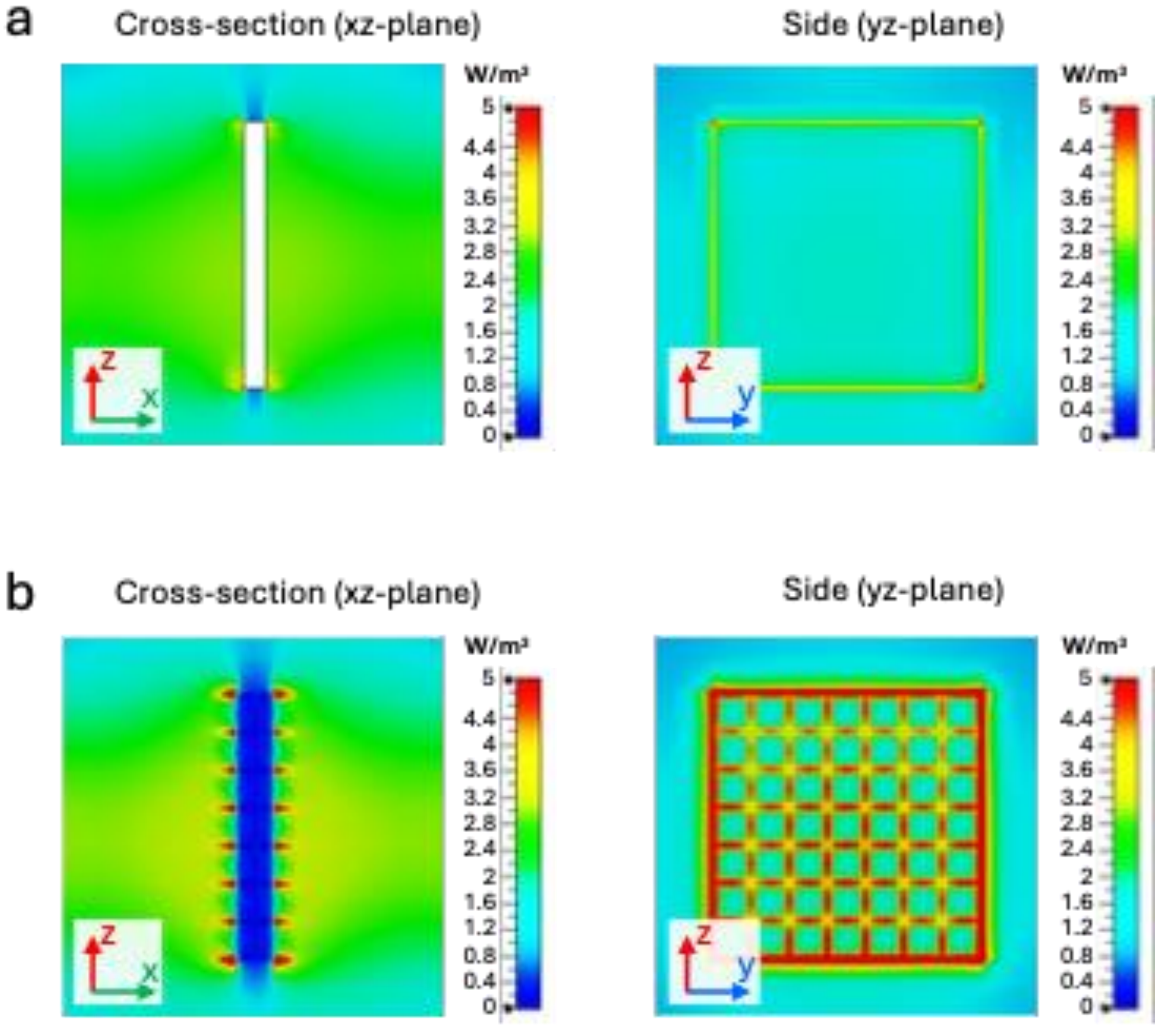

Figure S2. Simulated dielectric loss distribution of (a) plate structure and (b) mesh st ructure.

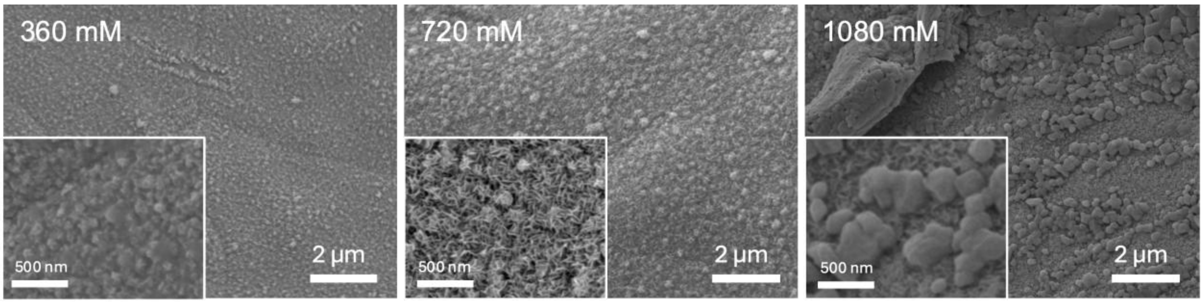

Figure S3. Morphology of the Fe-Ni_3S_2 catalyst synthesized on Ni foam with varying thiourea concentrations in a precursor solution of 30 mM $Ni(NO_3)_2 \cdot 6H_2O$ and 10 mM $Fe(NO_3)_3 \cdot 9H_2O$.

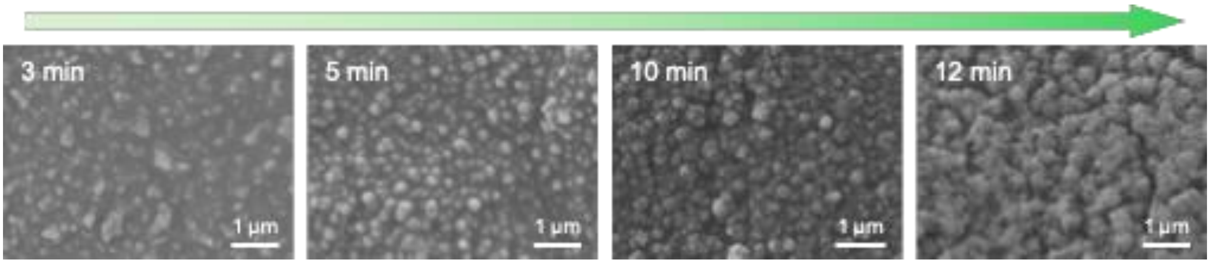

Figure S4. Time-dependent ex-situ SEM images showing the morphological evolution of Fe-Ni_3S_2 on a Ni foam.

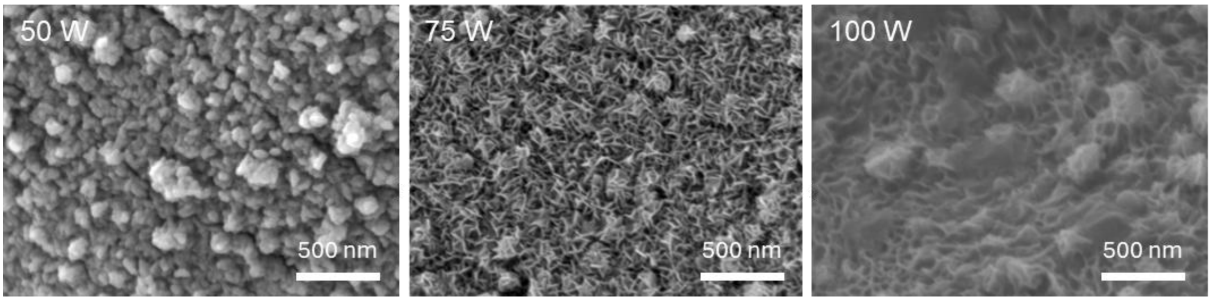

Figure S5. Morphology of the Fe-Ni_3S_2 catalyst synthesized on Ni foam with varying microwave power with a precursor solution of 30 mM $Ni(NO_3)_2·6H_2O$, 10 mM $Fe(NO_3)_3·9H_2O$ and 720 mM thiourea.

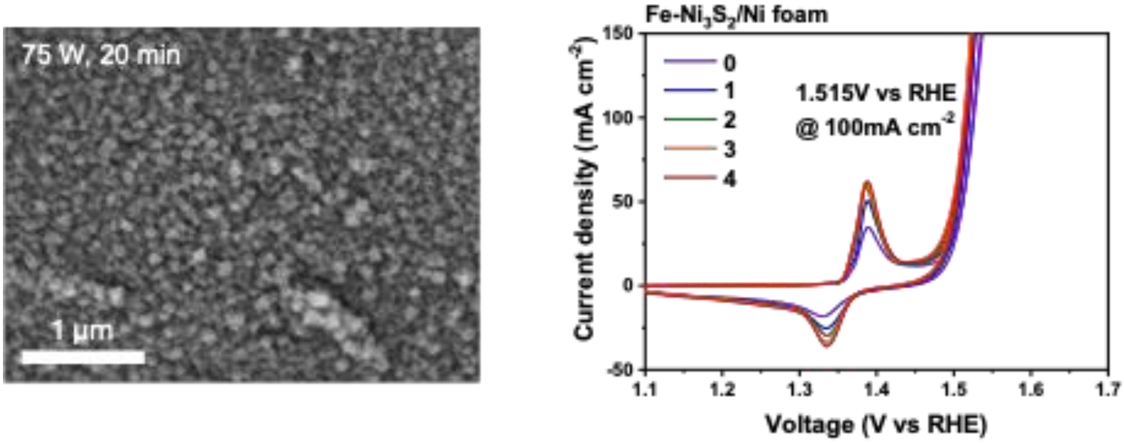

Figure S6. Structural and electrochemical characterization of the Fe-Ni_3S_2/Ni foam synthesized for 20 min.

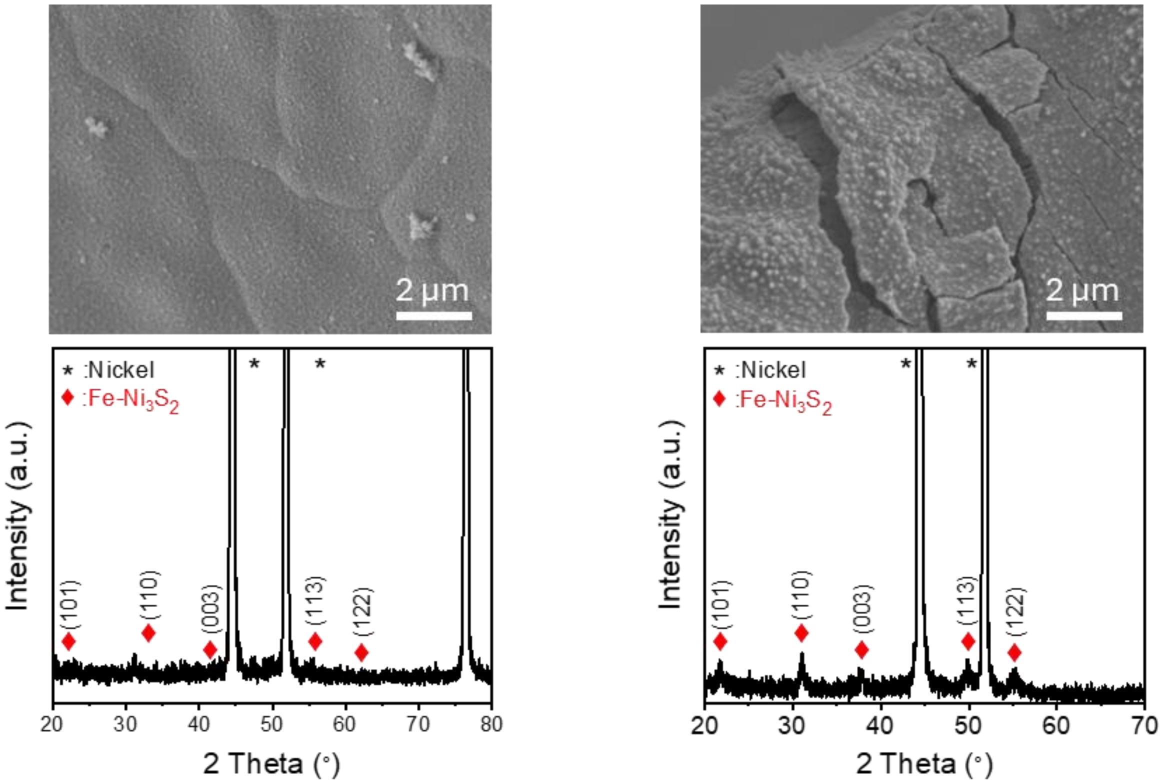

Figure S7. Morphology and crystallinity of the Fe-Ni_3S_2 catalyst synthesized on Ni fo am using solutions at half and double the concentration of the optimized precursor sol ution.

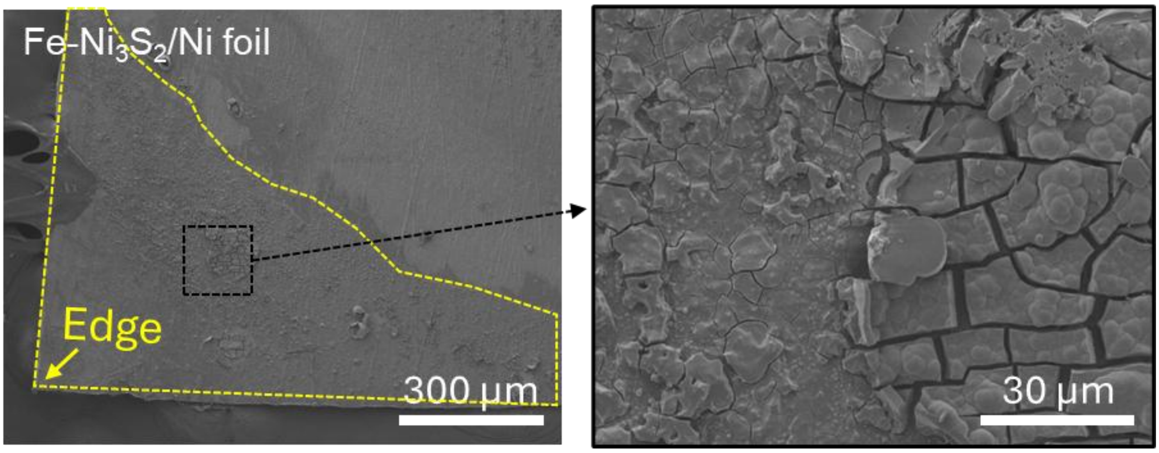

Figure S8. A low-magnification FE-SEM image of the Fe-Ni_3S_2 catalyst synthesized o n a Ni foam substrate under optimized conditions.

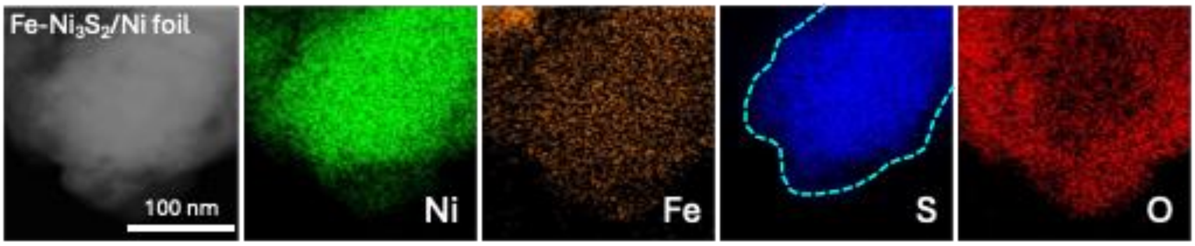

Figure S9. EDS elemental maps of Fe-Ni_3S_2 synthesized on Ni foil.

Sample	Ni (atomic %)	Fe (atomic %)	S (atomic %)	O (atomic %)
**Fe-Ni_3S_2/Ni foil**	43.80	5.67	30.95	19.58
**Fe-Ni_3S_2/Ni foam**	49.71	5.70	38.87	5.71

Table S2. Comparison of the quantitative elemental analysis for Fe-Ni_3S_2 catalysts synthesized on Ni foam versus Ni foil.

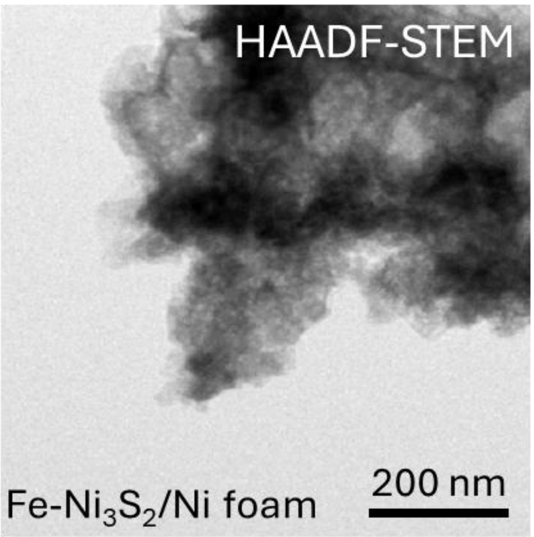

Figure S10. Low-magnification STEM image of Fe-Ni_3S_2 on Ni foam

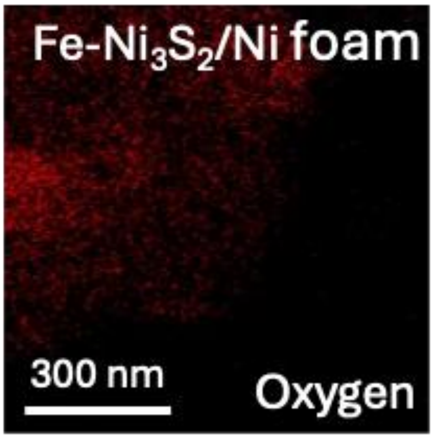

Figure S11. EDS oxygen maps of Fe-Ni_3S_2 synthesized on Ni foam

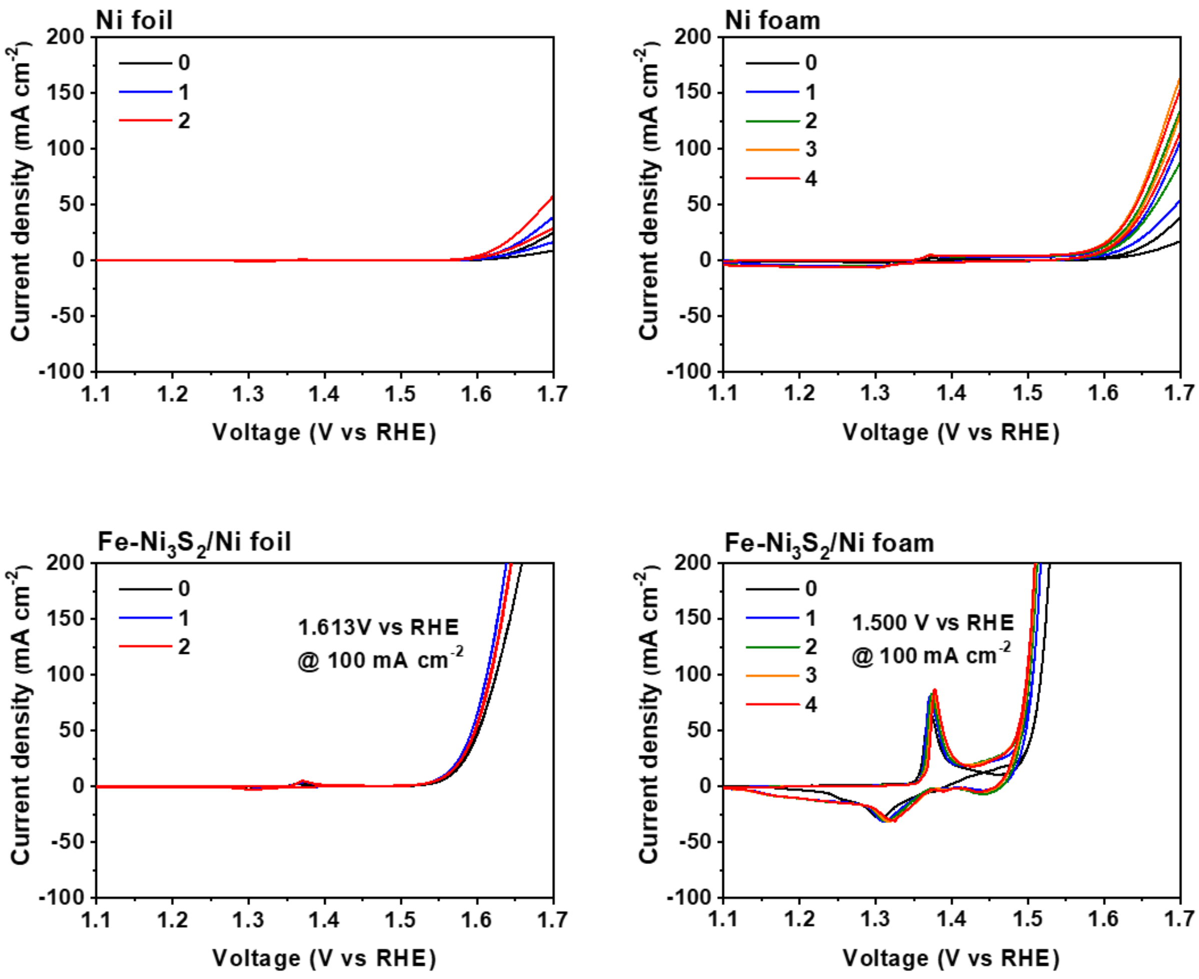

Figure S12. Catalytic activation of the Ni foil and foam, Fe-Ni_3S_2/Ni foil and Fe-Ni_3S_2/Ni foam by cyclic voltammetry.

Materials	Method	Synthesis condition	Overpotential (mV) at 100 mA cm^{-2}	Ref.
Fe-Ni_3S_2/Ni foam	Hydrothermal	12 h (160 °C) + 1 h (80 °C)	230	[8]
Fe-Ni_3S_2/Ni foam	Hydrothermal	6 h (150 °C)	290	[9]
Fe-Ni_3S_2/Ni foam	Hydrothermal	6 h (120 °C) + 8 h (100 °C)	249	[10]
Fe-NiS_2/Ni foam	Furnace	2 h (270 °C)	293	[11]
Fe-Ni_3S_2/Ni foam	Hydrothermal + Furnace	18 h (400 °C) + 30 min (400 °C)	287	[12]
NiFe LDH/$Ni(OH)_2$ /Ni foam	Laser + Microwave	15 min (532nm) + 1 min	~ 440	[13]
MnS_2/Co_4S_3/Ni_3S_2 /Ni foam	Hydrothermal	7 h (140 °C)	304	[14]
Fe-doped Ni_3S_2/FeS_2 /Ni foam	Hydrothermal	12 h (150 °C)	230	[15]
a-Ni_3S_2/Cu/Ni foam	Electrodeposition	15 min (RT)	370	[16]
NiFe-LDH/S-$NiMoO_4$ /Ni foam	Hydrothermal + Electrodeposition	5 h (150 °C) + 45 min (RT)	273	[17]
Fe-Ni_3S_2/Ni foam	Hydrothermal	15 h (160 °C)	295	[18]
FeOOH/Ni_3S_2 /Ni foam	Hydrothermal + Etching	6 h (150 °C) + 5 min (RT)	268	[19]
Fe-Ni_3S_2/Ni foam	Microwave	15 min	270	**This work**

Table S3. Comparison of OER overpotential and synthesis time with recently reported Ni, Fe-based catalysts.

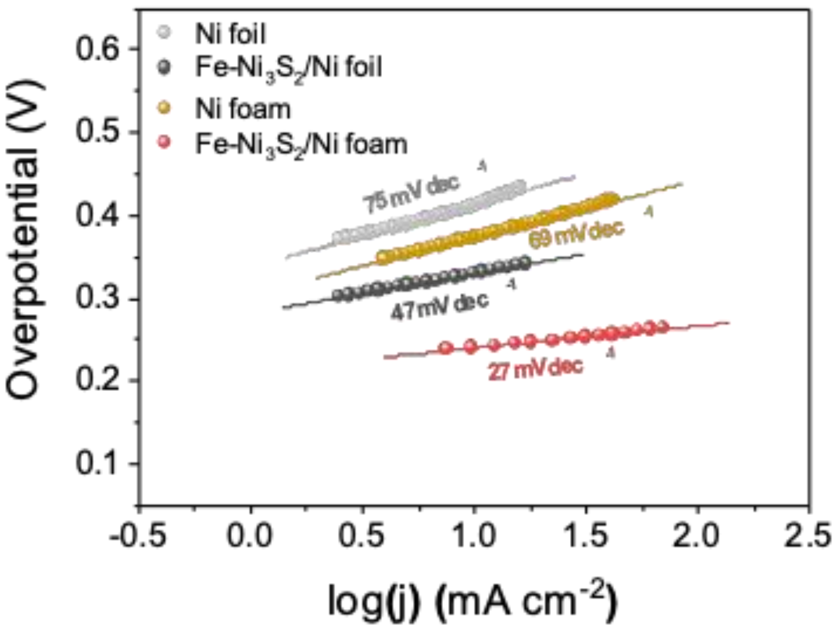

Figure S13. Tafel plots for the OER on Ni foil and foam, Fe-Ni_3S_2/Ni foil and Fe-N i_3S_2/Ni foam.

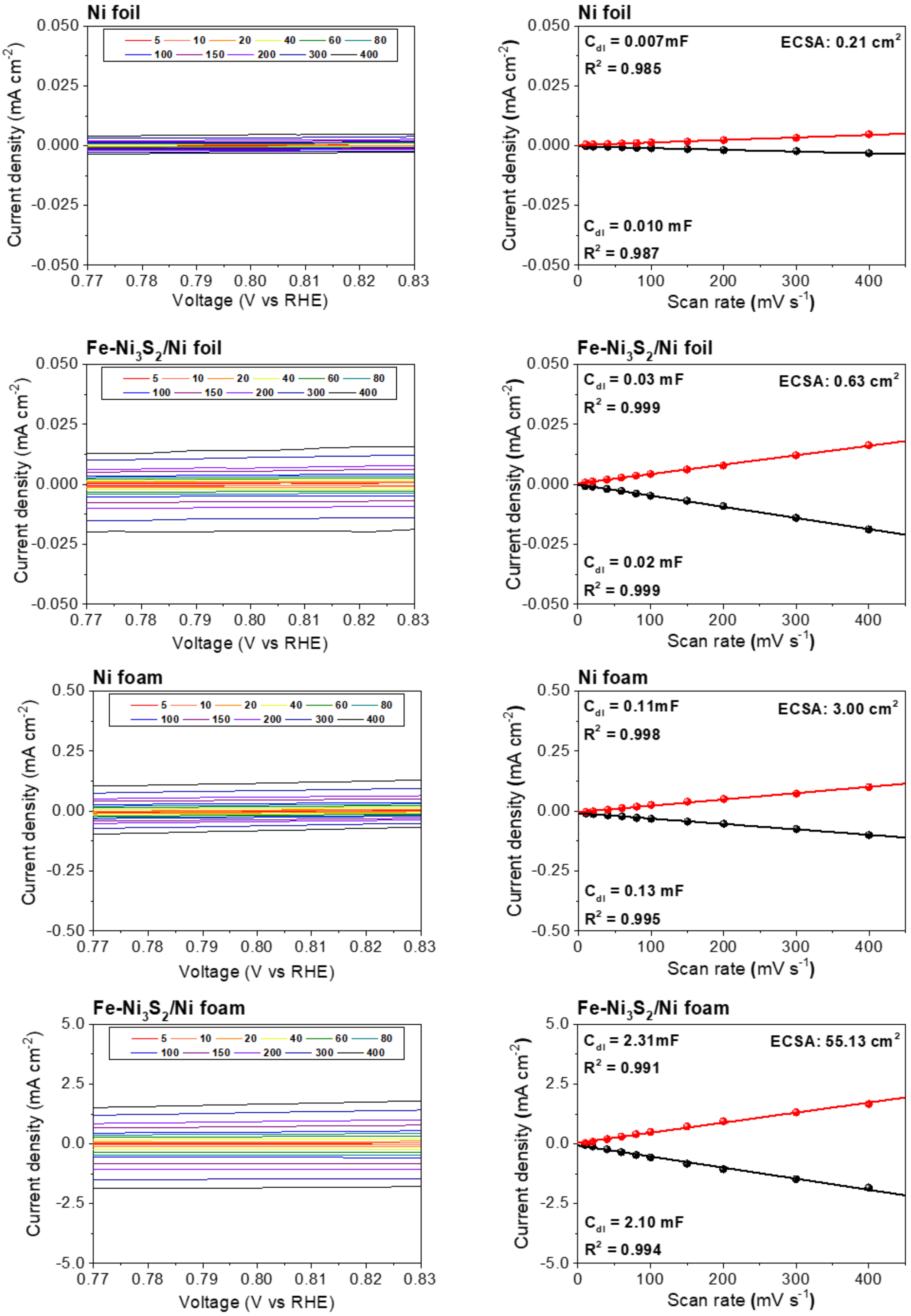

Figure S14. Double-layer capacitance (C_{dl}) for estimating the electrochemically active surface area in 1 M KOH. (a), (c), (e), (g) Cyclic voltammograms collected within a non-Faradaic potential window at various scan rates. (b), (d), (f), (h) Cathodic (black circles) and anodic (red circles) charging currents at 0.80 V vs RHE plotted as a fun ction of scan rate. C_{dl} was determined as the average of the absolute values of the sl opes obtained from linear fits to the cathodic and anodic datasets.

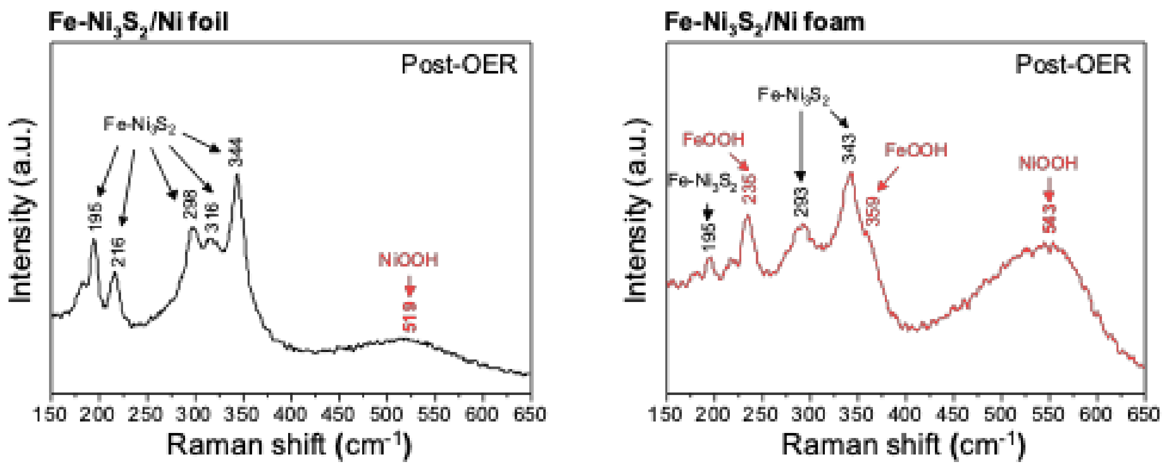

Figure S15. Raman spectroscopy of Fe-Ni_3S_2/Ni foil and Fe-Ni_3S_2/Ni foam after OER.

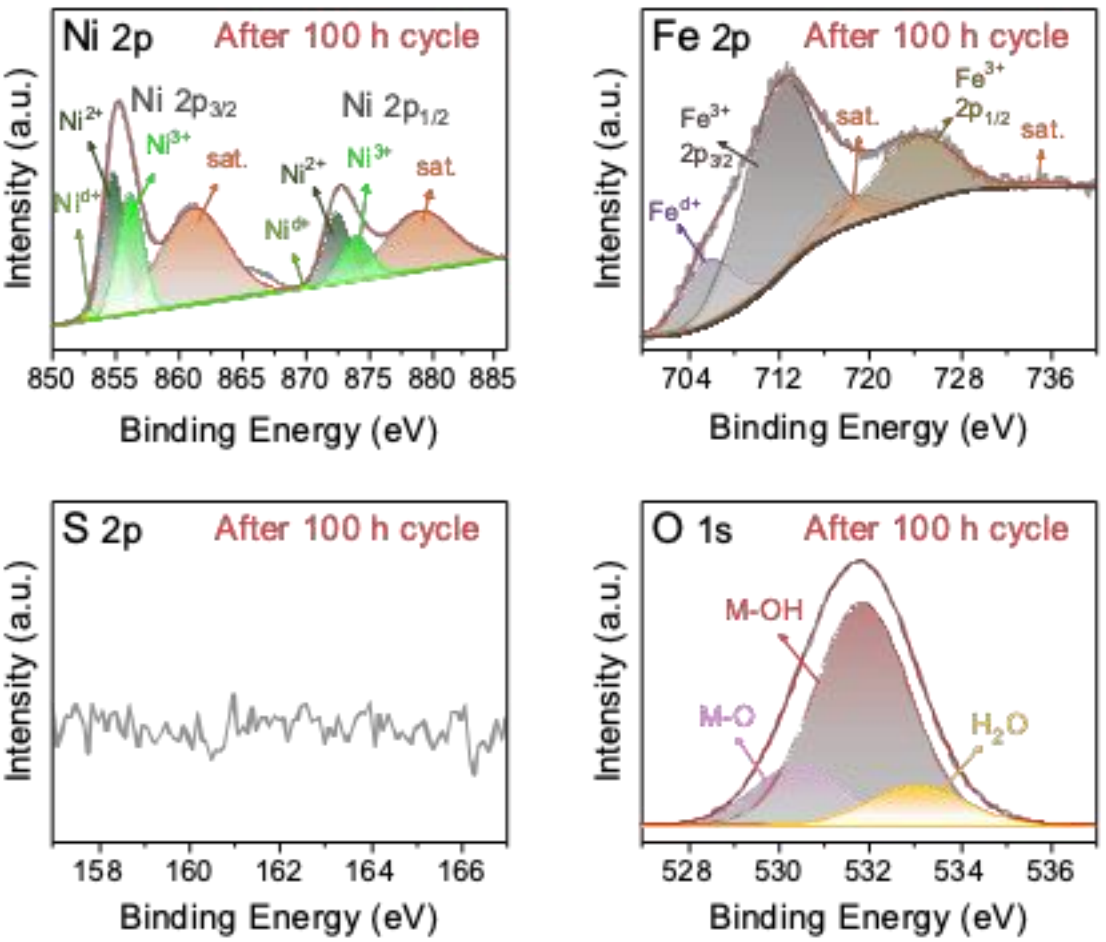

Figure S16. XPS spectra of the Ni 2p, Fe 2p, S 2p, and O 1s for Fe-Ni_3S_2/Ni foam after the 100 hours durability test.

References

(1) B. Zhang, X. Zheng, O. Voznyy, et al., "Homogeneously Dispersed Multimetal Oxygen-Evolving Catalysts," *Science* 352, no. 6283 (2016): 353–358. https://doi.org/10.1126/science.aaf1525

(2) L. Xia, B. F. Gomes, W. Jiang, et al., "Operando-Informed Precatalyst Programming towards Reliable High-Current-Density Electrolysis," *Nature Materials* 24, no. 5 (2025): 753–761. https://doi.org/10.1038/s41563-025-02128-7

(3) C. C. L. McCrory, S. Jung, J. C. Peters, and T. F. Jaramillo, "Benchmarking Heterogeneous Electrocatalysts for the Oxygen Evolution Reaction," *Journal of the American Chemical Society* 135, no. 45 (2013): 16977–16987. https://doi.org/10.1021/ja407115p

(4) Y. T. Kim, P. P. Lopes, S. A. Park, et al., "Balancing Activity, Stability and Conductivity of Nanoporous Core-Shell Iridium/Iridium Oxide Oxygen Evolution Catalysts," *Nature Communications* 8, (2017): 1449. https://doi.org/10.1038/s41467-017-01734-7

(5) S. Geiger, O. Kasian, M. Ledendecker, et al., "The Stability Number as a Metric for Electrocatalyst Stability Benchmarking," *Nature Catalysis* 1, no. 7 (2018): 508–515. https://doi.org/10.1038/s41929-018-0085-6

(6) B. J. Lee, S. M. Jung, G. Yu, et al., "Highly Active and Stable Al-Doped NiFe Self-Supported Oxygen Evolution Reaction Electrode for Alkaline Water Electrolysis," *ACS Catalysis* 15, no. 2 (2025): 1123–1134. https://doi.org/10.1021/acscatal.4c04393

(7) H. Shin, S. M. Jung, Y. J. Lim, et al., "Highly Efficient and Durable Ammonia Electrolysis Cell Using Zirfon Separator," *Advanced Science* 12, no. 7 (2025): 2500579. https://doi.org/10.1002/advs.202500579

(8) J. Zhu, W. Chen, S. Poli, et al., "Nanostructured Fe-Doped Ni3S2 Electrocatalyst for the Oxygen Evolution Reaction with High Stability at an Industrially-Relevant Current Density," *ACS Applied Materials & Interfaces* 16, no. 43 (2024): 58520–58535. https://doi.org/10.1021/acsami.4c09821

(9) D. Li, W. Wan, Z. Wang, et al., "Self-Derivation and Surface Reconstruction of Fe-Doped Ni3S2 Electrode Realizing High-Efficient and Stable Overall Water and Urea Electrolysis," *Advanced Energy Materials* 12, no. 41 (2022): 2201913. https://doi.org/10.1002/aenm.202201913

(10) G. Zhang, Y. S. Feng, W. T. Lu, et al., "Enhanced Catalysis of Electrochemical Overall Water Splitting in Alkaline Media by Fe Doping in Ni3S2 Nanosheet Arrays," *ACS Catalysis* 8, no. 6 (2018): 5431–5441. https://doi.org/10.1021/acscatal.8b00413

(11) G. Xiong, H. Deng, Y. Chen, et al., "Synergistic Fe-Doping and S-Vacancy Engineering in NiS2 for High-Performance Water Splitting," *ACS Catalysis* 15, no. 3 (2025): 13948–13957. https://doi.org/10.1021/acscatal.5c02496

(12) M. Shi, Z. Wan, L. Liu, et al., "Constructing a Homojunction of Fe-Ni3S2 as a Highly Efficient Electrocatalyst for the Oxygen Evolution Reaction," *Sustainable Energy & Fuels* 9, no. 5 (2025): 2500–2509. https://doi.org/10.1039/D5SE00171D

(13) D. H. Lee, R. Kerkar, D. Arumugam, et al., “Interfacial Charge Transfer Modulation in Laser-Synthesized Catalysts for Efficient Oxygen Evolution,” *Journal of Materials Chemistry A* 12, no. 46 (2024): 30269–30278. https://doi.org/10.1039/D4TA06794K

(14) H. Gao, Z. Xu, S. Lin, Y. Sun, and L. Li, “Construction of a Three-Phase MnS2/Co4S3/Ni3S2 Heterostructure for Boosting Oxygen Evolution,” *Langmuir* 40, no. 41 (2024): 21077–21085. https://doi.org/10.1021/acs.langmuir.4c02475

(15) N. S. Gultom, C. H. Li, D. H. Kuo, and H. Abdullah, “Single-Step Synthesis of Fe-Doped Ni3S2/FeS2 Nanocomposites for Highly Efficient Oxygen Evolution Reaction,” *ACS Applied Materials & Interfaces* 14, no. 35 (2022): 39917–39926. https://doi.org/10.1021/acsami.2c08246

(16) Q. Wu, H. Li, J. Wang, et al., “Unveiling Electronic Regulation Mechanism in Amorphous Ni3S2/Crystalline Cu Heterointerface for Alkaline Overall Water Splitting,” *Advanced Functional Materials* (2025): 2415501. https://doi.org/10.1002/adfm.202515501

(17) H. Wang, L. Chen, L. Tan, et al., “Electrodeposition of NiFe-Layered Double Hydroxide Layer on Sulfur-Modified Nickel Molybdate Nanorods for Highly Efficient Seawater Splitting,” *Journal of Colloid and Interface Science* 613, (2022): 349–358. https://doi.org/10.1016/j.jcis.2022.01.044

(18) H. Song, X. Xiong, J. Gao, et al., “Unveiling the Promotion of Fe in Ni3S2 Catalyst on Charge Transfer for the Oxygen Evolution Reaction,” *Small* 20, no. 46 (2024): 2404060. https://doi.org/10.1002/smll.202404060

(19) M. Chen, Y. Zhang, J. Chen, et al., "In Situ Raman Study of Surface Reconstruction of FeOOH/Ni3S2 Oxygen Evolution Reaction Electrocatalysts," *Small* 20, no. 19 (2024): 2309371. https://doi.org/10.1002/smll.202309371
